# Neuronal Apoptotic Bodies Facilitate Japanese Encephalitis Virus Infection and Pathogenicity

**DOI:** 10.1002/jev2.70366

**Published:** 2026-09-01

**Authors:** Chuan Zeng, Xiaowei Nan, Ting Wang, Junyao Xiong, Shuo Zhu, Chengjie Yang, Bibo Zhu, Youhui Si, Shengbo Cao, Jing Ye

**Affiliations:** ^1^ National Key Laboratory of Agricultural Microbiology Huazhong Agricultural University Wuhan Hubei China; ^2^ Hubei Hongshan Laboratory Wuhan Hubei P. R. China; ^3^ Frontiers Science Center for Animal Breeding and Sustainable Production College of Veterinary Medicine Huazhong Agricultural University Wuhan Hubei P. R. China; ^4^ The Cooperative Innovation Center for Sustainable Pig Production Huazhong Agricultural University Wuhan Hubei China

**Keywords:** ApoBDs, JEV, microglia, neuroinflammation, neurons, pathogenicity

## Abstract

Japanese encephalitis (JE) caused by Japanese encephalitis virus (JEV) infection remains one of the major types of viral encephalitis. The mechanisms underlying JEV infection and pathogenesis have not been fully elucidated, resulting in the absence of specific therapies for JE. Neuronal apoptosis is a well‐established outcome of JEV infection; however, its secondary contributions to JE pathogenesis remain largely uncharacterized. Here, we demonstrate that apoptotic bodies (ApoBDs) derived from JEV‐infected neurons package infectious JEV particles, facilitating viral transmission between neurons, as well as between neurons and microglia. Further investigations revealed that microglia predominantly internalize the ApoBDs via phagocytosis and dynamin 2‐dependent endocytosis. Moreover, lipids, rather than nucleic acids or proteins, are the key pro‐inflammatory constituents of ApoBDs. Subsequent studies demonstrated that ApoBDs activate microglia through the TLR2/TLR4–NF‑κB signalling. *In vivo* experiments showed that JEV‐loaded ApoBDs elicit significantly more severe neuroinflammation and pathological lesions compared to ApoBD‐free virions. These findings underscore the non‐negligible contribution of neuron‐derived ApoBDs to JEV pathogenesis, providing valuable insights for the development of novel therapeutic strategies against JE.

## 1 Introduction

Japanese encephalitis (JE) represents a significant public health burden as an acute neuroinvasive disease endemic across Asia and the Western Pacific regions, including China, India, Japan, and Southeast Asia (Campbell et al. 2011). It constitutes one of the leading etiologies of viral encephalitis in both paediatric and adult populations within these areas (Solomon et al. 2000; Wang et al. 2007). Global estimates indicate approximately 68,000 JE cases annually, with associated fatalities ranging from 13,600 to 20,400 (Sharma et al. 2021), underscoring its substantial morbidity and mortality.

Japanese encephalitis virus (JEV) enters the mammalian bloodstream through mosquito bites. Subsequent viremia facilitates systemic spread, allowing the virus to invade multiple organs—particularly the liver and spleen—and ultimately enabling it to cross the blood‐brain barrier (BBB) to infiltrate the central nervous system (CNS) (Turtle and Solomon 2018). Within the CNS, JEV preferentially targets neurons and induces neuronal apoptosis (Kitidee et al. 2023). Furthermore, JEV neuroinvasion provokes the activation of resident microglia and astrocytes, resulting in the secretion of proinflammatory cytokines and chemokines (Turtle and Solomon 2018; Yang et al. 2004). This neuroinflammatory cascade further leads to neuronal damage and impairs the integrity of the BBB, accompanied by neurological symptoms and even death (Chen et al. 2010; Ghoshal et al. 2007). Due to the lack of specific antiviral therapies, reliance on preventive strategies is necessary, primarily through vaccination and vector control.

Extracellular vesicles (EVs) are heterogeneous, membrane‐bound nanoparticles, ranging from ∼30 nm to several micrometers, secreted by virtually all cell types. Based on their biogenesis pathways, EVs are broadly classified into three main categories: exosomes (derived from multivesicular bodies), microvesicles (shed directly from the plasma membrane), and apoptotic bodies (ApoBDs). ApoBDs are generated during the execution phase of apoptosis through extensive plasma membrane blebbing and fragmentation (Urbanelli et al. 2019). Apoptotic stimuli trigger the formation of distinct membrane protrusions, including microtubule spikes, apoptopodia, and beaded apoptopodia, which subsequently detach to yield ApoBDs (Atkin‐Smith et al. 2015; Moss et al. 2006; Poon et al. 2014). These ApoBDs encapsulate diverse cellular components, such as cytosolic proteins, organelles (e.g., mitochondria and endoplasmic reticulum), and nuclear material (DNA and RNA) (Bergsmedh et al. 2006). The surface exposure of phosphatidylserine serves as a critical “eat‐me” signal for phagocytic clearance. Crucially, viruses can hijack the apoptotic process to establish infection. For instance, African swine fever virus (ASFV) induces apoptosis to generate ApoBDs that encapsulate intact virions, which not only facilitates efficient intercellular spread but also shields the virus from host immune defenses (Gao et al. 2023a). Similarly, ApoBDs produced by monocytes infected with influenza A virus (IAV) enhance viral proliferation while stimulating the host to generate robust innate and adaptive immune responses (Atkin‐Smith et al. 2020). This represents a sophisticated mechanism for enhancing viral dissemination and immunoevasion.

Currently, while the role of ApoBDs in viral transmission is increasingly recognized across various viruses, its potential involvement in the neuropathogenesis of JEV remains largely unexplored. In this study, our results demonstrate that ApoBDs derived from JEV‐infected neuronal cell line (N2a) harbour infectious JEV and significantly facilitate the rapid proliferation and dissemination of the virus. Furthermore, microglia predominantly uptake ApoBDs via phagocytosis and dynamin 2‐dependent endocytosis, which, in turn, induces robust pro‐inflammatory responses. Lipids are the principal ApoBD‑associated components that elicit microglial activation, and this response is mediated through the canonical TLR2/TLR4–NF‑κB axis. We also provide evidence that ApoBDs with equivalent JEV genomic RNA levels exhibit enhanced neuronal damage and greater lethality *in vivo* compared to free virions. These findings deepen our understanding of the pathogenic mechanisms underlying JEV infection and offer novel insight into therapeutic development for JE.

## 2 Materials and Methods

### 2.1 Cell Lines and Viral Stock

Mouse neuroblastoma cells (N2a), mouse microglial cells (BV2), and baby hamster kidney cells (BHK‐21) were cultured in DMEM (Sigma Aldrich, USA), supplemented with 10% fetal bovine serum (FBS), 100 IU/mL penicillin and 100 mg/mL streptomycin (P/S) at 37°C with 5% CO_2_. The JEV P3 strain (GenBank accession no. U47032.1) was propagated and passaged on BHK‐21 cells and stored in our laboratory. The JEV‐EGFP recombinant strain was kindly provided by professor Bo Zhang from the Wuhan Institute of Virology, China.

### 2.2 Isolation and Culture of Primary Neurons and Microglia

Primary embryonic cortical/hippocampal neurons were prepared as previously described with modifications (Qi et al. 2021). Culture plates were pre‐coated with 100 µg/mL poly‐L‐lysine (P2636, Sigma) and subsequently washed twice with sterile H_2_O. Cortex and hippocampi were isolated from fetal brains at embryonic days 17 to 18 (E17–E18). After removing the meninges and blood vessels, the tissue was minced and subjected to digestion with 0.125% trypsin at 37°C for 20 min, followed by gentle trituration with a pipette. The resulting cell suspension was filtered through a 70 µm nylon strainer into a 50 mL conical tube and centrifuged at 1000 rpm for 6 min. The supernatant was discarded, and the cell pellet was resuspended in DMEM supplemented with 5% (v/v) FBS before cell counting.

For the culture of primary neurons, 3×10^5^ cells/well were seeded on 24‐well plates for flow cytometry detection. After 3 hours (h) of incubation, the medium was replaced with Neurobasal Medium (21103049, Thermofisher) supplemented with 0.1% (v/v) P/S and 2% B27 supplement (17504044, Thermofisher), and the cells were maintained in a humidified incubator with 5% CO_2_ at 37°C.

For the culture of primary microglia, 8 to 10 × 10^6^ cells/flask were plated into T‐75 flasks. Over the first 6 days, the medium was replaced with DMEM/F12 supplemented with 10% FBS and 0.1% (v/v) P/S every 3 days. On day 12, the flasks were placed on a shaker at 1000 rpm for 30 min, and the floating cells were collected from the medium. The resulting cells, which comprised over 90% microglia, were utilized for subsequent downstream experiments.

For the mixed culture of primary neurons and microglia, 1.5×10^4^ primary microglia were seeded onto primary neurons that had been pre‐cultured in 24‐well plates for 4 days, with JEV inoculation performed 1–2 days following co‐culture initiation.

### 2.3 Caspase‐Glo 3/7 Assay

Caspase‐3/7 activity was measured using the Caspase‐Glo 3/7 Assay System (G8090, Promega) according to the manufacturer's instructions. Briefly, N2a and primary neurons were seeded in sterile white opaque 96‐well plates and cultured overnight at 37°C with 5% CO_2_ prior to JEV infection, with ABT‐263‐treated cells as positive controls. At 36 and 48 h post‐infection (hpi) for N2a cells, and at 24, 36, and 48 hpi for primary neurons, an equal volume of freshly Caspase‐Glo 3/7 working reagent was added to each well. Plates were gently shaken for 30 s and incubated at room temperature (RT) in the dark for 30 min to stabilize luminescent signals, after which the luminescence was measured using a calibrated microplate luminometer (Victor Nivo 3S, USA).

### 2.4 Plaque Assay

The viruses were serially diluted in a 10‐fold manner using DMEM, followed by inoculation on BHK‐21 cells in 24‐well plates. After 1 h of incubation at 37°C, the supernatants were removed, and the cells were washed three times with PBS. Subsequently, the cells were overlaid with 1 mL DMEM containing 1.75% sodium carboxymethyl cellulose (CMC) and 1% FBS. Following incubation for 5 days at 37°C, the cells were fixed with 10% formaldehyde and stained with a 0.6% crystal violet solution. Eventually, the visible plaques were counted, and the viral titers (PFU/mL) were calculated.

### 2.5 Western Blotting

Samples were harvested and incubated in lysis buffer (Beyotime Biotechnology, Shanghai, China) containing protease inhibitor for 30 min on ice, and the supernatants were collected after centrifuging at 12,000 rpm for 10 min at 4°C. After the supernatant was quantified via the Coomassie Brilliant Blue (CBB) method, 5× protein loading buffer was added, and the mixture was heated at 100°C for 10 min to ensure protein denaturation. After resolution by SDS‐PAGE, the protein lysates were transferred to methanol‐activated PVDF membranes. Following blocking with 2% bovine serum albumin (BSA) in TBST for 2 h at RT, the membranes were incubated overnight at 4°C with primary antibodies: cleaved caspase‐3 (Asp175) rabbit mAb (9661, CST), Lamin‐B1 rabbit mAb (AC057, Abclonal), Histone H3 rabbit mAb (AC070, Abclonal), CD47 rabbit mAb (A28498, Abclonal), HMGB1 rabbit mAb (A19529, Abclonal), β‐Actin mouse mAb (66009, Proteintech), β‐Tubulin rabbit mAb (A12289, Abclonal), GAPDH mouse mAb (BM1623, Boster), Phospho‐NF‐κB p65 (Ser536) rabbit pAb (310013, Zenbio), NF‐κB p65 rabbit pAb (380172, Zenbio), Phospho‐IκB alpha (Ser32/Ser36) rabbit pAb (340776, Zenbio), IκB alpha rabbit mAb (R380682, Zenbio), or mouse monoclonal antibodies (mAbs) against JEV structural proteins generated in our laboratory, including anti‐envelope (E), pre‐membrane (prM), capsid caspid (C), and non‐structural protein 5 (NS5) mAbs, followed by incubation with the appropriate secondary antibodies (BOSTER, USA). Finally, the protein bands were visualized with Tanon ECL Chemiluminescent Substrate (P180‐501, Tanon) and chemiluminescence imaging system (Tanon, China).

### 2.6 Cytotoxicity Assay

Primary microglia (2×10^4^ cells/well) and BV2 cells (3×10^4^ cells/well) were seeded in 96‐well plates. Test substances at varying concentrations were then added to each well of monolayer cells. Following 8 h of incubation at 37°C, the supernatant was aspirated, and medium supplemented with 10% cell counting kit‐8 reagent (CCK‐8; BMU106, Abbkine) was added to each well. After an additional incubation for 0.5–2 h, absorbance at 450 nm was measured with a microplate reader. Cell viability was calculated as: [(OD_450_(drug) − OD_450_(blank)) / (OD_450_(control) − OD_450_(blank))] × 100%.

### 2.7 Apoptosis Detection

For fluorescence detection, N2a cells were washed with Annexin V‐binding buffer and incubated with mCherry‐conjugated Annexin V (C1069S, Beyotime) for 15 min prior to three washes. Subsequently, cell nuclei were stained with Hoechst 33342 at RT for 30 min. The samples were then observed under a fluorescence microscope.

For flow cytometry analysis, N2a cells or primary neuron‐microglia mixed cultures were harvested, washed with ice‐cold PBS, and resuspended in 1× Annexin V‐binding buffer. The cells were then stained with Annexin V‐FITC (AT101, Multi Sciences) and Zombie NIR (423105, BioLegend) for 15 min at RT. Apoptotic cells were quantified using a flow cytometer, and the data were analyzed with FlowJo software.

### 2.8 Isolation of ApoBDs, ApoBD‐Free Supernatant, and Dead Cells

The ApoBDs, ApoBD‐free supernatant, and dead cells were isolated from JEV‐infected N2a cells as previously reported (Gao et al. 2023a; Phan et al. 2018). In brief, the supernatant of N2a cells infected with JEV at a multiplicity of infection (MOI) of 1 was collected at 48 hpi and centrifuged at 300 ×*g* for 10 min. The dead cell pellets were resuspended in DMEM, while the resulting supernatant was transferred to a new tube and centrifuged at 3000 × *g* for 20 min. The pellet from the second centrifugation was referred to as ApoBDs, while the supernatant containing multivesicular bodies and exosomes was defined as the ApoBD‐free supernatant (ApoBD‐free sup). ApoBDs from N2a cells treated with 0.5 µM Staurosporine (STS) for 10 h or 20 µM ABT‐263 for 18 h were isolated following the same procedure.

### 2.9 Purification of Free Virions

Supernatant from JEV‐infected N2a cells was collected and centrifuged at 500 ×*g* for 5 min to pellet dead cells. The resulting supernatant was then centrifuged at 2000 ×*g* for 10 min to eliminate large cellular debris and partial ApoBDs. Following this, the supernatant was centrifuged at 10,000 ×*g* for 30 min to remove small vesicles. Then sample was initially concentrated via ultracentrifugation at 150,000 ×*g* for 1.5 h in a Beckman ultracentrifuge using an SW32Ti rotor. The viral pellet was resuspended in an appropriate volume of PBS. Using an SW41Ti rotor, resuspended viral particles were gently layered onto a sucrose gradient (70%, 55%, 35%, and 20%) along the tube wall via syringe, followed by ultracentrifugation at 150,000 ×*g* for 2.5 h. The free viral fraction localized between the 55% and 70% sucrose gradient bands. After discarding the liquid above the viral layer, the viral particles were gently aspirated with a 200 µL pipette and transferred to a new ultracentrifuge tube. Sufficient PBS was added to wash away impurities, and re‐ultracentrifugation was performed to further concentrate the virus. The supernatant was discarded, and the pellet was resuspended in PBS, aliquoted into equal volumes, and stored at −80°C.

### 2.10 Precipitation and Dilution Assay

The assay procedure was adapted from a previously established protocol (Gao et al. 2023b). Both ApoBDs and ApoBD‐free sup collected from JEV‐infected N2a cells were diluted in 10 mL DMEM and subjected to centrifugation at 3000 ×*g* for 20 min. After centrifugation, 9 mL of the upper supernatant was gently aspirated. A small aliquot of the remaining 1 mL was used for viral titer analysis, while the remainder underwent 10‐fold serial dilution, which was repeated for four rounds.

### 2.11 RNA Extraction and Quantitative Reverse‐Transcriptional PCR (RT‐qPCR)

Total RNA was extracted using MagZol reagent (R4801‐02, Magen). Subsequently, the first‐strand cDNA was synthesized using ABScript III RT Master Mix with gDNA Remover (RK20429, ABclonal), according to the manufacturer's recommendations.

To determine the mRNA levels of host genes, RT‐qPCR was performed using 2X Universal SYBR Green Fast qPCR Mix (RK21203, ABclonal) on a ViiA 7 Real‐Time PCR System (Applied Biosystems). The relative mRNA levels of each gene were calculated and normalized to those of *β‐actin* using the 2^(−ΔΔCt) method.

To quantify the JEV genomic RNA level, TaqMan RT‐qPCR was performed using primers and a probe targeting the JEV E gene. A standard curve was constructed using 10‐fold serial dilutions of the JEV stock solution. The viral RNA level was calculated based on the established standard curve. All primers and the probe are listed in Table 1.

**TABLE 1:** Primer pairs used in this study.

Primer name	Sequences (5’‐3’)	Application
*Ccl5‐F*	TTGTCACTCGAAGGAACCGC	RT‐qPCR (SYBR Green)
*Ccl5‐R*	AGAGCAAGCAATGACAGGGAA	
*Il‐1β‐F*	ATGGGCAACCACTTACCTATTT	
*Il‐1β‐R*	GTTCTAGAGAGTGCTGCCTAATG	
*Il‐6‐F*	CTTCCATCCAGTTGCCTTCT	
*Il‐6‐R*	CTCCGACTTGTGAAGTGGTATAG	
*β‐actin‐F*	GATATCGCTGCGCTGGTCG	
*β‐actin‐R*	CATTCCCACCATCACACCCT	
JEV‐C‐F	GGCTTTTATCACGTTCTTCAAGTTT	
JEV‐C‐R	TGCTTTCCATCGGCCTAAAA	
JEV‐NS5‐F	TGCTCAACGAGACCACCAACT	
JEV‐NS5‐R	GCCGTGCTCCATTGATTCTGTT	
JEV‐E‐F	CTGGTCCATAGGGAGTGGTTTC	RT‐qPCR (Taqman)
JEV‐E‐R	CTCCACGCTGTGCTCGAA	
Probe	TGACCTCGCTCTCCCCTGGACG	

### 2.12 Immunocytochemistry Assay

Cells were fixed with 4% paraformaldehyde for 10 min at room temperature. Subsequently, the cells were blocked and permeabilized with 1% BSA containing 0.1% Triton X‐100 for 30 min at RT. They were then incubated overnight at 4°C with JEV E mAb and cleaved Caspase‐3 (Asp175) rabbit mAb, followed by incubation with FITC‐conjugated goat anti‐mouse secondary antibody for 1 h. Nuclei were counterstained with 4',6‐diamidino‐2‐phenylindole (DAPI) for 10 min. Finally, the cells were observed and imaged under an inverted fluorescence microscope (Nikon, Germany).

### 2.13 Labelling and Tracing of ApoBDs in Vitro

ApoBDs were incubated with 10 µM CypHer5E NHS Ester (312961‐83‐4, MCE) for 2 h at 37°C. After washing twice with PBS, the labelled ApoBDs were resuspended in DMEM and added to recipient N2a or BV2 cells. 3 h later, the cells were fixed with 4% paraformaldehyde, followed by permeabilization with 0.1% Triton X‐100 for 10 min. After blocking with 1% BSA for 30 min, the cells were incubated with an anti‐JEV E mAb overnight at 4°C. Following three washes with PBS, the cells were incubated with an Alexa Fluor 555‐conjugated secondary antibody (Invitrogen) and FITC‐Phalloidin (40735ES75, Yeasen) for 1 h. Cell nuclei were stained with DAPI for 10 min. The stained samples were observed using a confocal laser scanning microscope (Nikon, Germany).

### 2.14 ApoBDs Uptake Assay

ApoBDs were stained with either 4 µM Dil (C1036, Beyotime) for 20 min at RT, or with 10 µM CypHer5E NHS Ester for 2 h at 37°C. After washing twice with PBS, the ApoBDs were resuspended in 2% FBS‐supplemented DMEM. To assess the efficacy of pharmacological inhibition on microglial phagocytosis of ApoBDs, BV2 cells or primary microglia were preincubated with cytochalasin D (22144‐77‐0, MCE), dynasore (304448‐55‐3, MCE), EIPA (1154‐25‐2, MCE), methyl‐β‐cyclodextrin (128446‐36‐6, MCE), pitstop2 (1419320‐73‐2, MCE), or corresponding vehicle for 1 h. The medium was then aspirated, and the cells were incubated with the labelled ApoBDs, with the same compounds re‐added at the original concentration to continue culturing at 37°C for 3 h. For flow cytometric analysis to quantify phagocytic efficiency, the cells were washed three times with PBS, harvested after trypsin digestion, and subjected to DAPI staining to exclude dead cells. To evaluate microglial phagocytic efficiency via fluorescent observation, cells were fixed with 4% paraformaldehyde. CD45 rabbit pAb (A24802, Abclonal) was then added and incubated overnight at 4°C. Following thorough washing with PBS, FITC‐conjugated secondary antibody was incubated for 1 h at RT, and counterstained with Hoechst 33342 for 10 min. Finally, observations were performed using a confocal laser scanning microscope.

To assess the role of ApoBDs in mediating JEV invasion into microglia, microglia were pretreated with each of the aforementioned inhibitors or vehicles. Subsequently, ApoBDs were added to the microglia, followed by incubation at 4°C for 1 h to suppress microglial endocytic activity. After discarding the supernatant, the cells were rinsed three times with PBS. Inhibitor‐containing medium was then re‐supplemented, and the cells were incubated at 37°C for 30 min to resume relevant cellular processes. Afterward, the supernatant was aspirated, and the cells were washed three additional times with PBS before adding MagZol reagent. Samples were collected and stored at −80°C until RNA extraction for the detection of intracellular JEV genome content.

### 2.15 Enzymatic, Detergent and MβCD Treatments of ApoBDs

ApoBDs were treated with various reagents, either individually or in combination, according to the following protocols. For Triton X‐100 treatment, ApoBDs were resuspended in 0.05% Triton X‐100, incubated at RT for 10 min, and washed twice with PBS. For nuclease treatment, ApoBDs were incubated with either 100 µg/mL RNase or 1000 U DNase I at 37°C for 1 h or 10 min followed by two PBS washes. For the combined treatment of Triton X‐100 and nucleases, the Triton X‐100 incubation and washing steps were performed first, followed sequentially by incubation with either DNase I or RNase. For protease treatment, ApoBDs were resuspended in a 200 µg/mL Proteinase K solution, incubated at 37°C for 15 min, and washed twice with PBS before use. The Proteinase K heated at 95°C for 10 min for inactivation served as a control. For MβCD treatment, ApoBDs were incubated in a 10 mM MβCD solution at 37°C for 1 h, followed by washes with PBS.

### 2.16 Immunoelectron Microscopy (IEM)

Both JEV‐infected N2a cells and ApoBDs were fixed with an immunoelectron microscopy fixative solution (G1124‐100ML, Servicebio) at 4°C for preservation. After washing three times with pre‑cooled PBS, the pellets were wrapped in 2% low‑melting‑point agarose (A8350, Servicebio) pre‑warmed to about 40°C. Dehydration was carried out sequentially in 30% ethanol at 4°C for 20 min, followed by 50%, 70%, 80%, 85%, 90%, 95% and two washes in 100 % ethanol at −20°C for 20, 20, 10, 10, 10, 10, 10 and 10 min, respectively. The samples were then infiltrated with LR White resin (14381‐UC, Haidebio) at 4°C in mixtures of ethanol:resin at ratios of 2:1 (1 h), 1:1 (3 h), 1:2 (17–20 h), followed by pure resin (17–20 h) and two changes of fresh pure resin (1 h each). For embedding, samples were placed in capsules containing pure resin and loosely capped. After evacuation under vacuum for 0.5–1 h, the caps were tightened and polymerization was performed in a low‑temperature UV polymerizer at −20°C for at least 48 h. Ultrathin sections 70–80 nm were cut using an ultramicrotome and collected on 150‑mesh nickel grids coated with Formvar film. For immunolabelling, grids were floated on ultrapure water for 5 min. After rinsing three times with TBS (G0015‐500ML, Servicebio), the grids were blocked with 1 % BSA in TBS for 30 min at RT. Primary antibodies were incubated overnight at 4°C. After returning to RT, grids were washed three times with TBS and incubated with secondary goat anti‐mouse IgG conjugated with 12‐nm gold and goat anti‐rabbit IgG conjugated with 4‐nm gold for 20 min at RT, then 1 h at 37°C, and finally 0.5 h at RT. After five washes with TBS and five with ultrapure water, grids were stained with 2 % uranyl acetate in saturated ethanol for 15 min in the dark, and then rinsed three times in 70 % ethanol and three times in ultrapure water. After rinsing, the grids were dried on filter paper and then further dried at 37°C for 10 min. Observation was conducted using transmission electron microscopy.

### 2.17 Enzyme‐linked Immunosorbent Assay (ELISA)

Cell culture supernatants were collected from treated cells and centrifuged at 500 ×*g* for 5 min to remove cellular debris. The resulting supernatants were stored at −80°C until analysis. The levels of inflammatory cytokines, including CCL5 (KE10017, Proteintech), IL‐1β (KE10003, Proteintech) and IL‐6 (RK00008, Abclonal), were quantified using commercial ELISA kits according to the manufacturers’ protocols.

### 2.18 Dynamic Light Scattering (DLS)

The size distribution of ApoBDs was determined using a Zetasizer system (Malvern Instruments, USA). Briefly, the diluted ApoBDs suspension (1:100 in PBS containing 0.2% BSA) was subjected to a monochromatic laser beam, and the size distribution was analyzed using Zetasizer software. The number of replicates for each measurement ranged from 13 to 19, depending on sample quality and the default settings of the DLS instrument.

### 2.19 Mice and Viral Infection

Four‐week‐old C57BL/6J mice were obtained from the Laboratory Animal Center of Huazhong Agricultural University, Wuhan, China. Mice were randomly assigned to three groups, among which, two groups were injected intracerebrally into the right hemisphere with ApoBDs or free virions (both diluted in 25 µL DMEM) from JEV‐infected N2a cells at an equivalent viral genomic level. The other group received an equal volume of DMEM was served as control. The mice were housed under standard laboratory conditions. Daily body weight measurements, general health assessments, and disease progression evaluations were performed for all groups until day 14. On day 7, the viral loads and distributions, levels of inflammatory cytokines, and pathologic changes in brain tissues were evaluated.

### 2.20 Histological and Immunofluorescent Staining

Mice were anesthetized with 1.5% sodium pentobarbital via intraperitoneal injection. After the mice entered a state of deep anaesthesia (confirmed by absence of pedal reflex and reduced respiratory rate), cardiac perfusion was performed using 20 mL of PBS. Brains were carefully harvested and bisected along the midline: the left hemisphere was fixed in 4% paraformaldehyde for subsequent paraffin embedding and frozen sectioning.

For hematoxylin‐eosin (HE) staining, brain paraffin sections were deparaffinized in xylene (2 × 5 min), followed by hydration in gradient ethanol (100%, 95%, 80%, 70%; 3 min each) and rinsing with distilled water. Nuclei were stained with hematoxylin for 10 min, differentiated in 1% hydrochloric acid‐ethanol for 30 seconds, then blued under running tap water for 5 min. Sections were counterstained with eosin for 1–3 min, then dehydrated in gradient ethanol (70%, 80%, 95%, 100%; 3 min each) and cleared in xylene (2 × 5 min). Finally, sections were mounted with neutral balsam and air‐dried at RT for subsequent microscopic examination.

For immunohistochemistry (IHC), deparaffinized and hydrated sections underwent antigen retrieval in 0.01 M citrate buffer (pH 6.0) at 95°C for 15 min, then cooled to RT naturally. Sections were blocked and permeabilized with 1% BSA containing 0.1% Triton X‐100 for 1 h, followed by incubation with Iba1 rabbit mAb (A19776, Abclonal) at 4°C overnight. Sections were washed with PBS and then incubated with HRP‐conjugated secondary antibody at 37°C for 1 h. Sections were developed with 3,3’‐Diaminobenzidine (DAB) chromogen until distinct brown signals appeared, then terminated with distilled water. Nuclei were lightly counterstained with hematoxylin for 30 seconds, followed by dehydration through gradient ethanol, clearing in xylene, and mounting with neutral balsam for subsequent microscopic examination.

For immunofluorescence (IF) staining, frozen brain sections were rinsed three times with PBS, followed by blocking and permeabilization with 1% BSA containing 0.1% Triton X‐100 for 1 h. Subsequently, sections were incubated overnight at 4°C with primary antibodies, including JEV‐E mAb, ASC rabbit mAb (A24165, Abclonal), NLRP3 rabbit pAb (A5652SP, Abclonal), IL‐1β rabbit mAb (A27676, Abclonal), cleaved caspase‐3 (Asp175) rabbit mAb, MAP2 Rabbit mAb (A22205, Abclonal), and Iba1 rabbit mAb, respectively. Following three PBS washes, sections were incubated with the corresponding secondary antibodies for 1 h at RT. Nuclei were counterstained with DAPI for 10 min. After three additional PBS washes, sections were mounted with an anti‐fade mounting medium and coverslipped. Images were acquired using a confocal laser scanning microscope.

### 2.21 Statistical Analysis

All data were analyzed using GraphPad Prism 9 (GraphPad Software, La Jolla, CA, USA). The measured values are presented as the mean ± standard deviation (SD) for *in vitro* experiments and as mean ± standard error of mean (SEM) for *in vivo* experiments. Statistical significance was analyzed using the two‐tailed unpaired Student's *t*‐test or one‐way ANOVA with subsequent multiple comparisons, as appropriate.

## 3 RESULTS

### 3.1 JEV Infection Induces Neuronal Apoptosis

**FIGURE 1:**
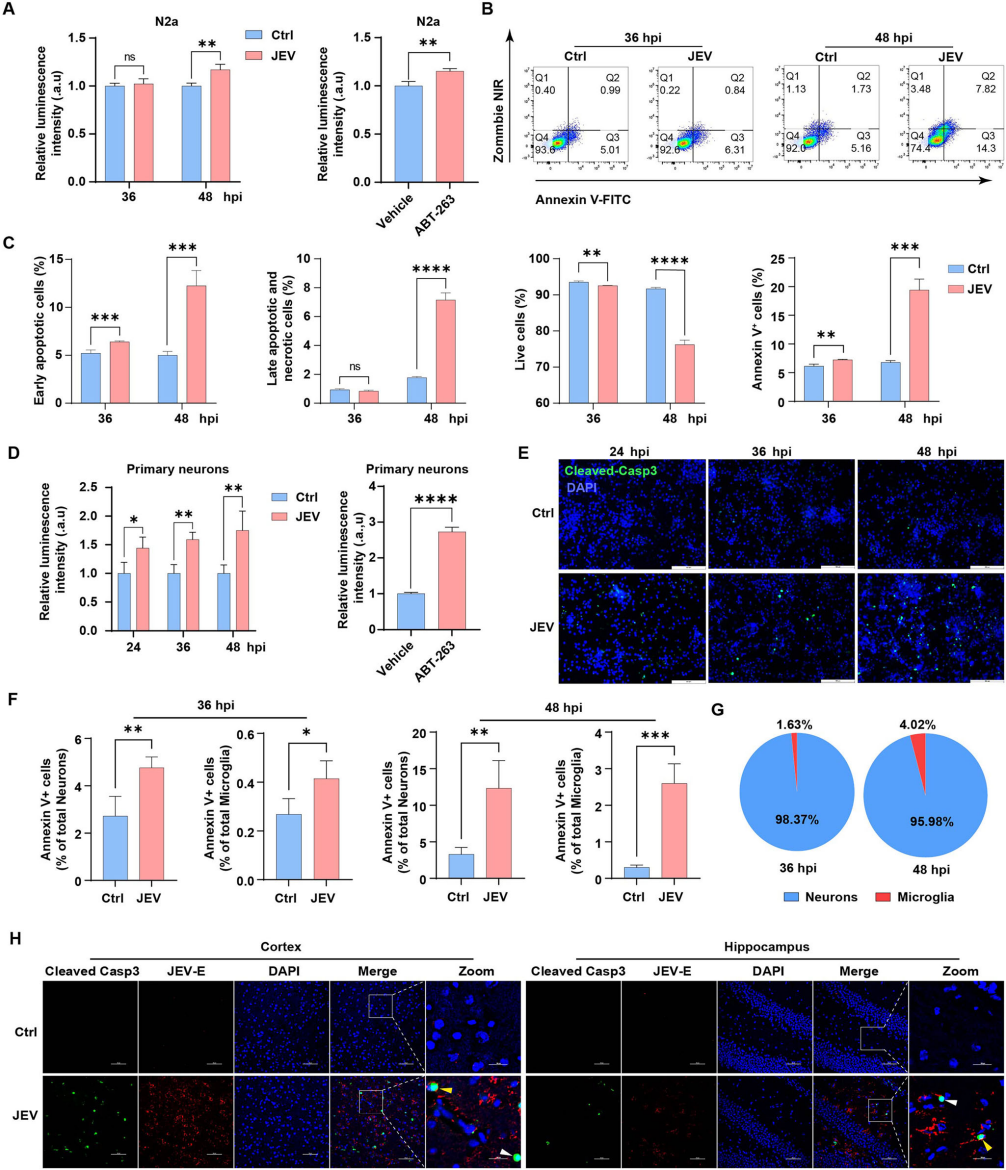
JEV infection induces neuronal apoptosis. (A–C) N2a cells were infected with JEV (MOI = 1) or treated with 20 µM ABT‐263. At 36 and 48 hpi or 18 h post‐treatment, the cells were harvested and subjected to apoptosis assessment using the Caspase‑Glo 3/7 assay (A). Flow cytometric analysis of apoptosis was conducted via Annexin V‐FITC and the viability dye Zombie NIR co‐staining (B). Quantification of the percentages of cells in each quadrant from (B) is shown (C). Q1 (Zombie NIR^+^/Annexin V^−^): naked nuclei; Q2 (Zombie NIR^+^/Annexin V^+^): late apoptotic/necrotic cells; Q3 (Zombie NIR^−^/Annexin V^+^): early apoptotic cells; and Q4 (Zombie NIR^−^/Annexin V^−^): live cells. (D–E) Primary mouse neurons were infected with JEV (MOI = 1) or treated with 5 µM ABT‑263. The cells were harvested at 24, 36 and 48 hpi, or 5 h post‐treatment and subjected to apoptosis detection using the Caspase‑Glo 3/7 assay (D). Immunofluorescence assay was performed using anti‐cleaved caspase‐3 antibody (green) with DAPI counterstaining for nuclei (blue). Scale bar: 100 µm (E). (F–G) Primary neurons and microglia co‐culture were infected with JEV (1 MOI) and harvested at 36 and 48 hpi. The proportions of Annexin V positive neurons and microglia within their respective subpopulations were analyzed by flow cytometry at 36 hpi (left) and 48 hpi (right), respectively (F). The proportions of Annexin V positive neurons and microglia in the total cell population were analyzed by flow cytometry at 36 hpi (left) and 48 hpi (right), respectively (G). (H) Frozen mouse brain sections harvested on day 7 after intraperitoneal challenge were subjected to immunofluorescence staining using an anti‐cleaved caspase‐3 antibody (green) and an anti‐JEV E antibody (red), and the cortical and hippocampal regions were presented. Yellow arrowheads mark JEV/cleaved caspase‐3 double‐positive cells, while white arrowheads denote JEV‐negative, cleaved caspase‐3‐positive cells. Scale bar: 100 µm or 500 µm (Enlarged views). Data are presented as mean ± SD of four biological replicates. Statistical analysis was performed using a two‐tailed unpaired Student's *t*‐test (A, C, D and F). *p < 0.05, **p < 0.01, ***p < 0.001, ****p < 0.0001.

To verify the JEV‐induced apoptosis in neurons, N2a cells were either infected or mock‐infected with JEV at an MOI of 1 or 5. Immunofluorescence analysis showed that the efficiency of viral infection peaked at 36 hpi and then gradually declined under both 1 and 5 MOI conditions (Figure S1A). Similar results were obtained from the viral growth curve, which illustrated the production of infectious virions in the cell supernatants at various time points (Figure S1B). Notably, cells infected with 5 MOI of JEV exhibited a significantly greater decrease in cell viability compared to those infected with 1 MOI (Figure S1C). To examine the occurrence of apoptosis in JEV‐infected N2a cells, the Caspase‐Glo 3/7 assay was employed, which quantifies the proteolytic activity of caspase‐3/7. At 48 hpi, JEV elicited a significant enhancement of the luminescence signal, similar to the effect observed with the BH3 mimetic ABT‐263, a well‐known inducer of cell apoptosis (Figure 1A). This observation was corroborated by immunostaining, which revealed extensive cleaved caspase‐3‐positive cells in JEV‐infected groups (Figure S1D). As phosphatidylserine that flips to the outer cell membrane when cells undergo apoptosis can be selectively bound by Annexin V (Fadok et al. 1992; Koopman et al. 1994; Vermes et al. 1995), we further assessed neuronal apoptosis using flow cytometry and fluorescence assays. Flow cytometric analysis revealed that JEV infection markedly elevated the percentage of Annexin V‐positive apoptotic cells, with the most prominent increase observed at 48 hpi (Figure 1B,C), as validated by Annexin V‐mCherry staining (Figure S1E). Although only early apoptosis was significantly enhanced at 36 hpi, JEV infection strongly induced both early and late apoptosis at 48 hpi, which was accompanied by a substantial reduction in the proportion of viable cells (Figure 1C). These findings confirm that JEV infection can induce apoptosis in N2a cells.

To further assess the pro‐apoptotic effect of JEV, primary mouse neurons were isolated and infected with JEV at an MOI of 1. The luminescence signals indicated a significant, time‐dependent upregulation of caspase 3/7 activity, which started as early as 24 hpi, with progressively stronger activation at 36 and 48 hpi (Figure 1D). Furthermore, treatment of primary neurons with ABT‐263 recapitulated the phenotype observed in N2a cells, inducing a marked elevation in caspase‐3/7 activity (Figure 1D). Immunofluorescence staining of cleaved caspase‐3 in primary neurons matched the temporal activation pattern observed in the Caspase‐Glo 3/7 luminescence assay, thereby suggesting JEV induces apoptosis in primary neurons as well (Figure 1E). Given the complex communication between neurons and other cell types, such as glial cells, within CNS, we further evaluated the ability of JEV to cause apoptosis in mixed cultures of primary neuron and microglia. The results demonstrated that JEV infection triggered apoptosis in both primary neurons and microglia, with neurons constituting the majority of apoptotic cells (Figure 1F,G). To validate JEV‐induced apoptosis in the CNS, we examined cleaved caspase‐3 expression in mouse brain sections. Cleaved caspase‐3 immunoreactivity was observed in both the cortex and hippocampus of JEV‐infected mice (Figure 1H). Importantly, the apoptotic cells included not only JEV‑infected cells but also uninfected cells, indicating a bystander effect of JEV‐induced cell death in the CNS.

### 3.2 Isolation and Characterization of ApoBDs From JEV‐Infected Neurons

**FIGURE 2:**
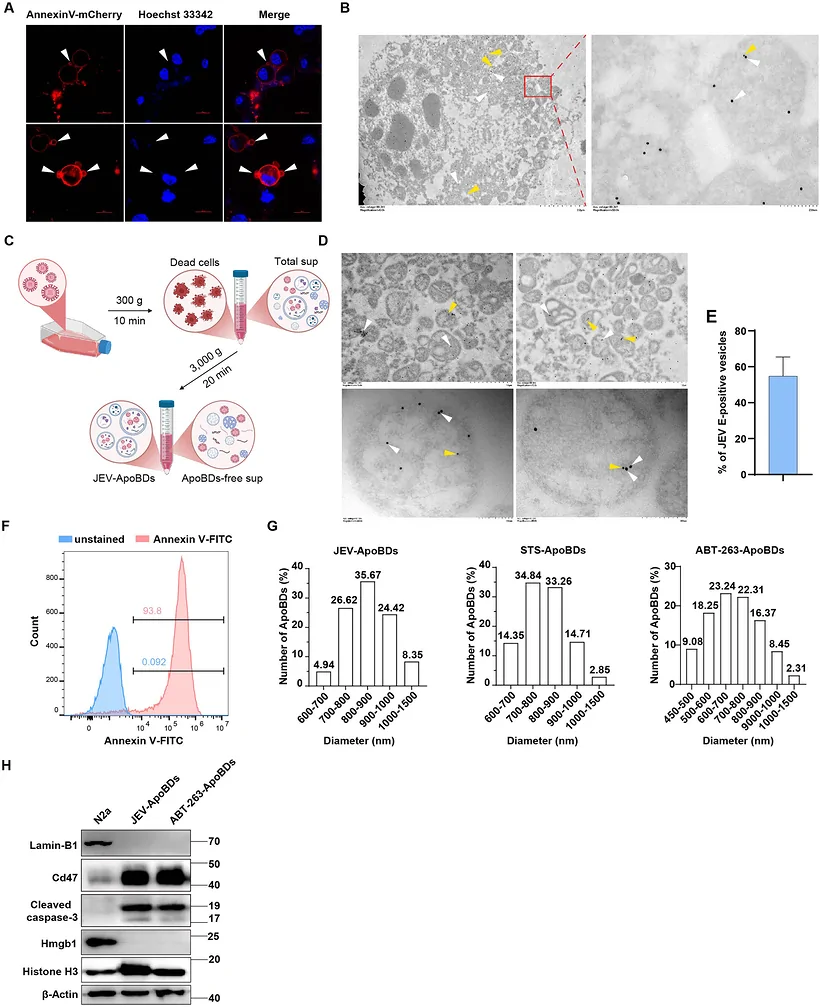
Isolation and characterization of ApoBDs derived from N2a Cells. (A) N2a cells infected with 1 MOI JEV for 48 h were stained with Annexin V‐mCherry and Hoechst 33342, and imaged via confocal microscopy. White arrowheads indicate protrusion vesicles. Scale bar: 10 µm. (B) IEM observation of N2a cells infected with JEV (1 MOI) at 48 hpi. White arrowheads indicate JEV particles (12 nm gold particles); yellow arrowheads indicate cleaved‐caspase 3 (4 nm gold particles). Scale bars: 2 µm (left panel) and 200 nm (right panel). (C) Schematic diagram of ApoBDs isolation. (D) IEM observation of purified ApoBDs. Scale bars: 2 µm (top panels) and 200 nm (bottom panels). (E) Quantification of JEV‑containing vesicles in (D) (n = 24 fields). (F) Flow cytometric assessment of the purity of Annexin V‐FITC‐labeled ApoBDs isolated from JEV‐infected N2a cells, presented as a ridgeline plot. (G) The size distributions of ApoBDs derived from JEV‐infected (JEV‐ApoBDs), STS‐stimulated (STS‐ApoBDs) and ABT‐263‐stimulated (ABT‐263‐ApoBDs) N2a cells were measured using DLS. (H) The protein markers of ApoBDs induced by ABT‐263 and JEV in N2a cells were analyzed by Western blotting. Schematic diagram in (C) was created using BioRender (https://www.biorender.com).

To evaluate whether JEV infection can trigger the formation of ApoBDs from neurons, N2a cells were incubated with mCherry‐conjugated Annexin V at 48 hpi. Annexin V‐positive protruding vesicles were observed on the surface of Annexin V‐labelled cells (Figure 2A). These budding structures closely resemble the classic apoptotic membrane blebs and protrusions documented in previous scanning electron microscopy (SEM) studies of apoptotic cells (Chang et al. 2000; Zhang et al. 2018), suggesting that JEV induces neurons to form pre‐ApoBD‐like vesicles. To substantiate this finding, IEM analysis was performed on JEV‐infected N2a cells. Dual immunogold labelling demonstrated the coexistence of JEV E protein (12 nm gold particles) and cleaved caspase‐3 (4 nm gold particles) within both the cytoplasm and shed vesicles (Figure 2B), providing direct evidence that these vesicles derived from apoptotic cells harbour viral particles.

Building on the above findings, we sought to isolate ApoBDs from the culture supernatant of JEV‐infected N2a cells using the differential centrifugation method established previously (Figure 2C) (Phan et al. 2018). IEM imaging showed that the majority of the isolated fractions were intact membrane‐bound vesicular structures, rather than irregular cellular debris (Figure 2D). Crucially, these purified vesicles retained both JEV E protein and cleaved caspase‐3. Quantitative assessment revealed that JEV E protein was detectable in more than half of these vesicles (Figure 2E). Moreover, the proportion of Annexin V‐positive vesicles exceeded 90%, as evidenced by flow cytometry assay (Figure 2F). To further characterize the isolated vesicles, we measured their particle size range using DLS. STS, a broad‐spectrum protein kinase inhibitor derived from Streptomyces staurosporeus, and ABT‐263 were employed as positive apoptotic stimuli. The diameter of vesicles derived from both JEV‐infected and STS‐treated N2a cells was greater than 500 nm, with the majority concentrated in the range of 700–1000 nm and a small fraction exceeding 1000 nm (Figure 2G). In comparison, more than 90% of vesicles from ABT‑263‑treated N2a cells were above 500 nm in size, whereas roughly 9% fell into the 450–500 nm range. The slight size variation among the three groups likely reflects differences in apoptotic signalling and membrane blebbing kinetics, yet all vesicles remained predominantly larger than 500 nm, consistent with the size hallmark of ApoBDs. Informed by prior reports on ApoBD markers (Atkin‐Smith et al. 2015; Chen et al. 2022; Garikipati et al. 2018), we next performed Western blot analysis to profile the molecular signatures of these isolated vesicles (Figure 2H). The nuclear envelope protein Lamin‐B1 and the damage‐associated nuclear protein Hmgb1 were nearly undetectable in both JEV‐ and ABT‐263‐induced vesicles, suggesting negligible contamination by intact nuclei or necrosis‐derived nuclear proteins. Conversely, the surface marker CD47 and nuclear Histone H3 exhibited obvious enrichment in these vesicle preparations. Cleaved caspase‐3 was exclusively detected in JEV‐ and ABT‐263‐induced vesicles, while being entirely absent in whole N2a cells, further confirming the apoptotic origin of these isolated vesicles. In conclusion, these results demonstrate the successful isolation and purification of ApoBDs from JEV‐infected N2a cells.

### 3.3 ApoBDs Facilitate Transmission of JEV Between Neurons and Between Neurons and Microglia

**FIGURE 3:**
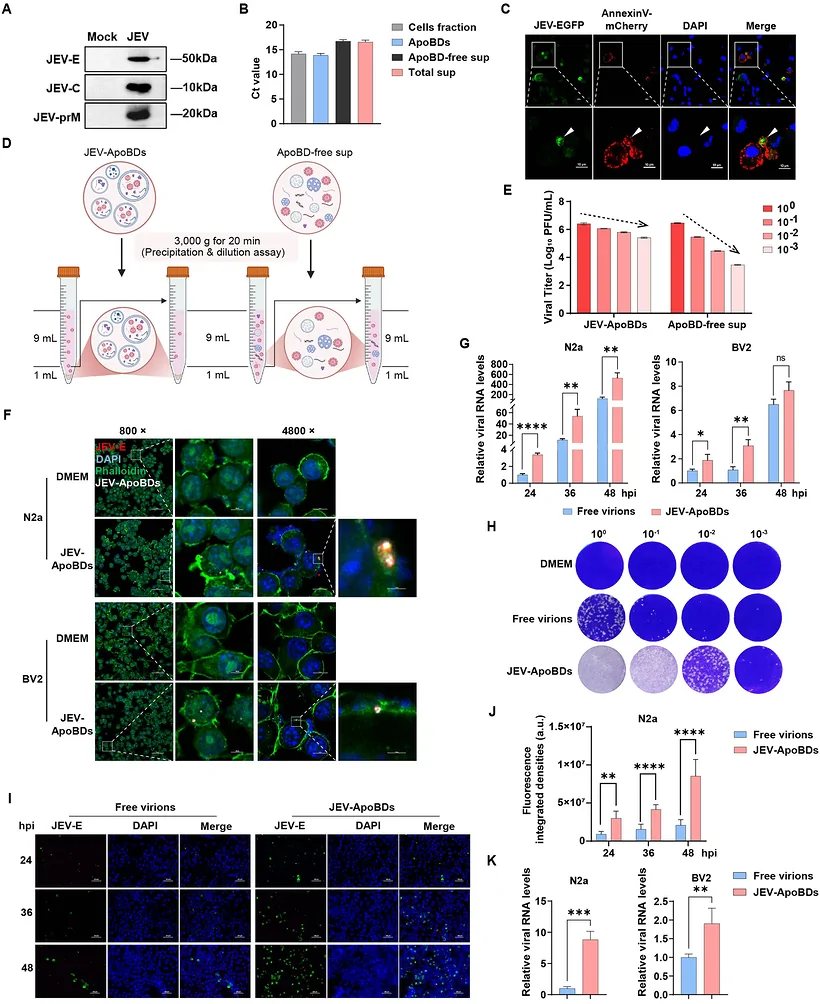
ApoBDs carrying infectious virus can be taken up by neural cells. (A) N2a cells were mock‐infected or infected with JEV at 1 MOI and then harvested for Western blot analysis using the indicated antibodies at 48 hpi. (B) N2a cell were infected with JEV (1 MOI) for 48 h, and the RNA levels of JEV E gene in different fractions, including N2a cells, JEV‐ApoBDs, total supernatant, ApoBD‐free supernatant, were quantified using RT‐qPCR. (C) N2a cells were infected with recombinant JEV‐EGFP at an MOI of 1. The cells were stained with Annexin V‐mCherry (red) and DAPI (blue) at 48 hpi. (D) Schematic diagram of parallel precipitation and dilution assay. (E) Following parallel precipitation and dilution, JEV‐ApoBDs and ApoBD‐free supernatant from JEV‐infected N2a cells were subjected to plaque assay to determine the viral titers across different dilutions. (F) CypHer5E‐labeled JEV‐ApoBDs were added to N2a or BV2 cells. After 3 h of incubation, the cells were fixed and stained with JEV E mAb, FITC‐Phalloidin and DAPI, and images were captured using a confocal microscope. Scale bars: 50 µm, 400 µm, 10 µm, and100 µm (from the left column to the right column). (G) Free virions and JEV‐ApoBDs with the same JEV genomic RNA levels were separately incubated with N2a and BV2 cells. After incubation for the specified time points, cells were harvested, and the relative intracellular JEV RNA levels were quantified via RT‐qPCR using the 2^(−ΔΔCt) method. (H) Plaque assay was performed on free virions and JEV‐ApoBDs with equivalent JEV genomic RNA levels. (I) The infectivity of virions and JEV‐ApoBDs with the same viral genomic levels in N2a cells was determined by immunofluorescence assay at 24, 36, and 48 hpi. (J) The fluorescence integrated intensity of JEV E protein in (I) was quantified. (K) Free virions and JEV‐ApoBDs with equivalent JEV genomic RNA levels were separately incubated with N2a and BV2 cells at 4°C for 1 h and then at 37°C for 30 min. The viral RNA invasion was assessed by RT‐qPCR using the 2^(−ΔΔCt) method. Data are presented as mean ± SD of at least three biological replicates. Statistical analysis was performed using a two‐tailed unpaired Student's *t*‐test (G, J, K). *p < 0.05; **p < 0.01; ***p < 0.001; ****p < 0.0001; ns, not significant. Schematic diagram in (D) was created using BioRender (https://www.biorender.com).

Given the detection of viral particles within the ApoBDs derived from JEV‐infected N2a cells, we subsequently examined the additional viral cargo in the purified ApoBDs. Western blot analysis ascertained the presence of JEV structural proteins, including E, prM, and C, within the ApoBDs (Figure 3A). Additionally, viral genomes were successfully detected in the ApoBDs through RT‐qPCR, with Ct values comparable to those of JEV‐infected N2a cells (Figure 3B). Furthermore, a recombinant virus expressing EGFP (JEV‐EGFP)—in which the EGFP gene was inserted downstream of the 38th N‐terminal residue of the capsid protein—was employed to infect N2a cells (Zhang et al. 2020), enabling the monitoring of JEV's dynamic trajectory. The results showed that the EGFP signal was encapsulated in protrusive, pre‐ApoBD‐like structures of Annexin V‐positive cells (Figure 3C), suggesting that JEV‐EGFP may be shed and released via ApoBDs as cell apoptosis proceeds.

To further clarify whether the ApoBDs derived from JEV‐infected neurons carry infectious virions, we assessed the infectivity of the ApoBDs. To eliminate the possibility of contamination from free virions, we performed parallel sedimentation and dilution assays on both isolated ApoBDs and ApoBD‐free supernatant (Figure 3D) (Gao et al. 2023b). Compared to the undiluted ApoBD‐free supernatant, undiluted ApoBDs exhibited a similar viral titer; however, serial dilution caused a proportional decrease in viral titer in the ApoBD‐free supernatant, whereas ApoBDs maintained a stable viral titer. This observation verifies that the infectivity of ApoBDs is independent of any extraneous free virions (Figure 3E). Collectively, these findings suggest that the ApoBDs produced by JEV‐infected N2a cells contain infectious virions.

Based on the above observations, we speculated that the ApoBDs carrying infectious virions may mediate intercellular transmission of JEV. Therefore, we utilized CypHer5E, a pH‐sensitive fluorescent probe, to visualize the endocytosis of EVs. ApoBDs isolated from JEV‐infected N2a cells were labelled with CypHer5E and then added to uninfected N2a and BV2 cells. The results showed that both cell types efficiently internalized the JEV‑loaded ApoBDs (Figure 3F). To compare the infectivity of JEV‐containing ApoBDs with that of free virions, these recipient cells were incubated with either ApoBDs produced by JEV‐infected N2a cells or sucrose gradient‐purified free virions (Figure S2A), both of which had equivalent viral genomic RNA abundances. The results demonstrated that, regardless of cell type or time point, the JEV carried by ApoBDs exhibited significantly higher infectivity compared to free virions. This was evidenced by higher intracellular viral genome levels (Figure 3G) and viral titer (Figure 3H), as well as enhanced infectious ratios (Figures 3I,J and S2B,C). To elucidate why JEV encapsulated by ApoBDs presents higher infectivity, we investigated the role of ApoBDs in viral internalization. N2a and BV2 cells were incubated with JEV‐containing ApoBDs or free virions at the same viral RNA levels at 4°C for 1 h before shifting to 37°C for 30 min. As expected, JEV carried by ApoBDs was internalized more efficiently by these cells (Figure 3K), compared to the free virions. These results demonstrate that ApoBDs facilitate viral internalization, thereby promoting viral transmission between neurons and between neurons and microglia.

### 3.4 Microglia Take up ApoBDs Produced by JEV‐Infected Neurons Through Phagocytosis and Dynamin 2‐Dependent Endocytosis

**FIGURE 4:**
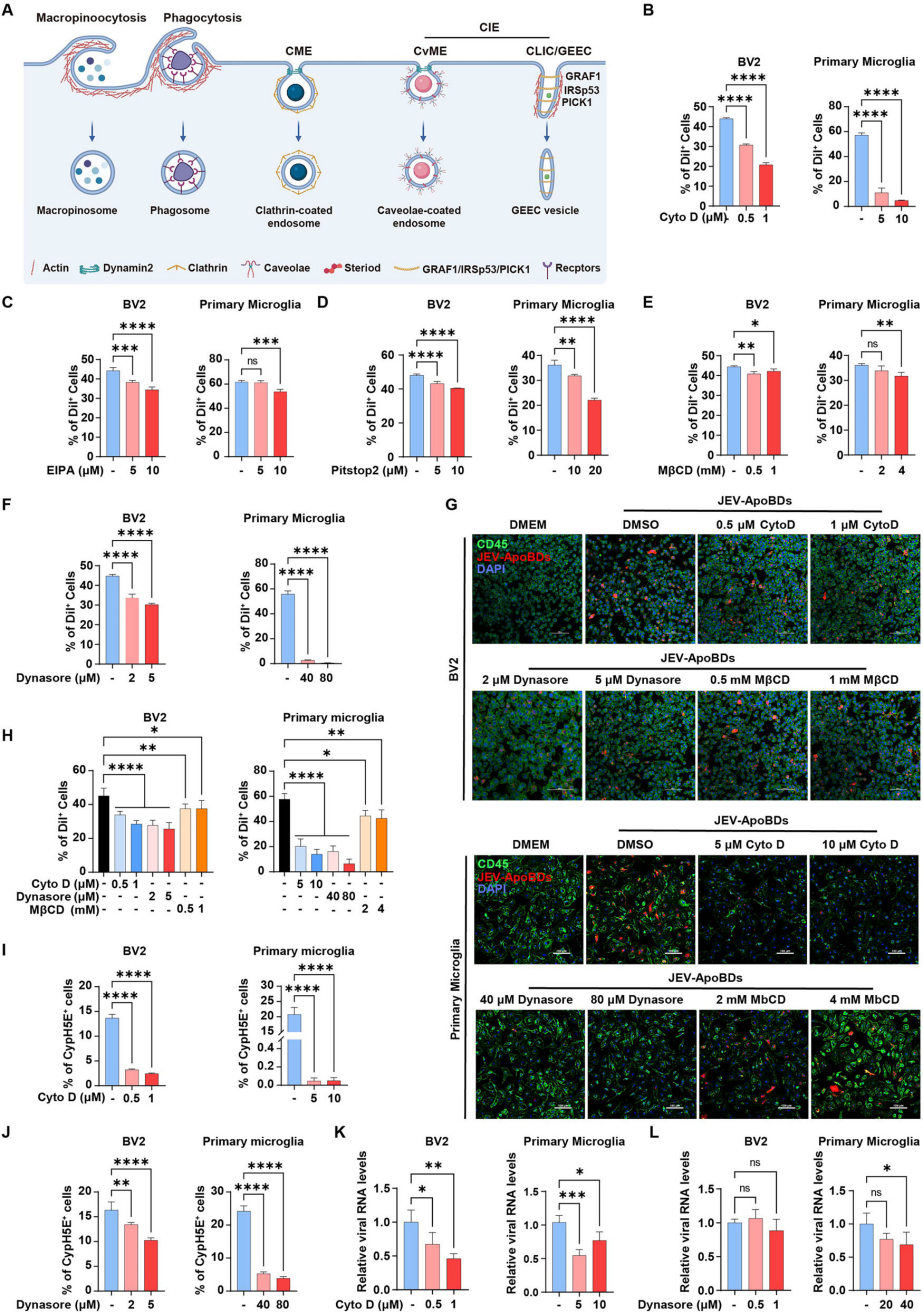
Pharmacological inhibition of ApoBDs uptake by microglia. (A) Schematic diagram of endocytosis pathways. CME: clathrin‐mediated endocytosis; CIE: clathrin‐independent endocytosis; CvME: caveolin‐mediated endocytosis; CLIC/GEEC: clathrin‐independent carriers/GPI‐AP‐enriched early endosomal compartments; GRAF1: GTPase regulator associated with focal adhesion kinase‐1; IRSp53: insulin receptor substrate protein of 53 kDa; PICK1: protein interacting with C kinase 1. (B–H) BV2 cells and primary microglia were pre‐incubated with the indicated inhibitors for 1 h. The medium was then replaced with inhibitor‐containing medium supplemented with Dil‐labeled JEV‐ApoBDs. After 3 h of incubation, these cells were analyzed by flow cytometry (B–F) or visualized using a confocal microscope. Scale bar: 100 µm (G). Quantification of endocytosis efficiency in (G) (H). (I–J) BV2 cells and primary microglia were treated as described in (B–F) but incubated with CypHer5E‐labeled JEV‐ApoBDs, followed by flow cytometric analysis. (K–L) BV2 cells and primary microglia were pre‐incubated with the indicated inhibitors for 1 h. After the medium was removed, the cells incubated with JEV‐ApoBDs for 1 h at 4°C, followed by washing and continued incubation at 37°C for 30 min. The intracellular viral RNA was quantified via RT‐qPCR using the 2^(−ΔΔCt) method. Data are expressed as mean ± SD from at least three biological replicates. One‐way ANOVA followed by Dunnett's post‐hoc test was used for statistical analysis, with significance defined as: ns, not significant; *p < 0.05; **p < 0.01; ***p < 0.001; ****p < 0.0001. Schematic diagram in (A) was created using BioRender (https://www.biorender.com).

Numerous studies have documented the uptake of EVs by recipient cells through various endocytosis pathways, including phagocytosis, macropinocytosis, clathrin‐mediated endocytosis (CME), and clathrin‐independent endocytosis (CIE) (Figure 4A). The pathways utilized are contingent on the subpopulations of EVs and the types of recipient cells (Doherty and McMahon 2009; Donaldson 2013; Feng et al. 2010; Grosse et al. 2005; Mulcahy et al. 2014). To identify the specific pathways through which microglia take up ApoBDs released by JEV‐infected neurons, we employed pharmacological inhibitors that selectively target distinct endocytic pathways at safe doses (Figure S3). Cytochalasin D (Cyto D), an inhibitor of actin polymerization that interferes with both phagocytosis and micropinocytosis (Mylvaganam et al. 2021), remarkably abrogated the internalization of ApoBDs produced by JEV‐infected N2a cells in both BV2 cells and primary microglia (Figure 4B). To further delineate the specific actin‐dependent endocytic pathway responsible for mediating ApoBDs uptake, EIPA, a classic macropinocytosis inhibitor, was employed. The results indicated that macropinocytosis plays a limited role in ApoBDs uptake (Figure 4C), providing preliminary evidence that phagocytosis, rather than micropinocytosis, functions as the major pathway mediating this process. In addition, the CME pathway was found to be partially involved in the uptake of ApoBDs derived from JEV‐infected neurons, whereas the CIE pathway appeared negligible, as evidenced by the respective application of pitstop2, a clathrin‐targeting inhibitor, and methyl‐β‐cyclodextrin (MβCD), a lipid raft disruptor (Figure 4D,E). Importantly, dynamin 2, which acts as the “key scission motor” for endocytic vesicle fission from the plasma membrane, was shown to be indispensable for ApoBDs uptake by primary microglia, as treatment with 40 µM dynasore, a dynamin inhibitor, profoundly suppressed this uptake, and this inhibition was further potentiated at 80 µM (Figure 4F). Similarly, dynamin 2 also contributed to ApoBDs uptake by BV2 cells. These findings were corroborated by fluorescence imaging, which showed that treatment with the aforementioned inhibitors decreased the accumulation of ApoBDs within microglia, most notably in Cyto D and dynasore groups (Figures 4G,H and S4A,B). Concurrently, both inhibitors potently reduced the percentage of microglia containing ApoBDs in acidic compartments (Figure 4I,J). We next assessed the role of phosphatidylserine exposed on the ApoBD surface in regulating this uptake process. The specific blockade of these surface lipids markedly abrogated the microglial engulfment of ApoBDs (Figure S4C). Collectively, these data support the notion that phagocytosis and dynamin 2‐dependent endocytic pathways synergistically mediate the uptake of ApoBDs from JEV‐infected neurons by microglia.

To explore whether the endocytic patterns responsible for ApoBDs uptake are associated with JEV internalization, viral genomic RNA was detected in microglia incubated with ApoBDs derived from JEV‐infected N2a cells in the presence or absence of Cyto D or dynasore. As expected, Cyto D treatment significantly attenuated the viral genome load in both BV2 cells and primary microglia following exposure to ApoBDs (Figure 4K). Dynasore prominently decreased viral genome content in primary microglia, with a weak reducing effect observed in BV2 cells (Figure 4L). These results are consistent with the inhibitory effects of both inhibitors on microglial capacity to internalize ApoBDs (Figure 4B,F,I, and J), which shed light on the mechanism underlying the enhanced infectivity of JEV contained within ApoBDs.

### 3.5 ApoBDs Produced by JEV‐Infected Neurons Elicit Robust Inflammatory Responses in Microglia

**FIGURE 5:**
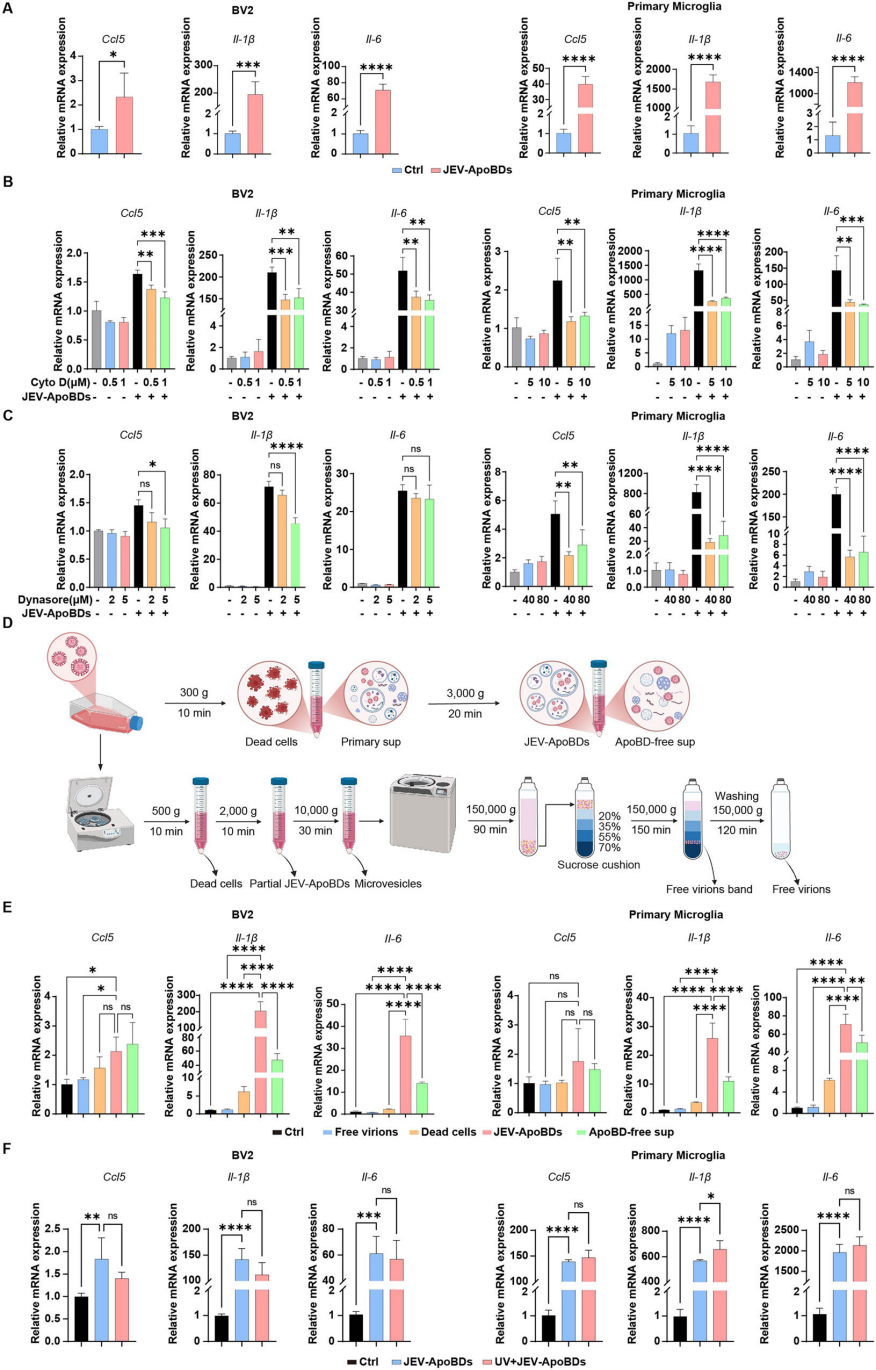
ApoBDs produced by JEV‐infected neurons induce the inflammatory responses in microglia. (A) BV2 cells and primary microglia were incubated with or without JEV‐ApoBDs for 6 h. The relative mRNA levels of *Ccl5*, *Il‐1β* and *Il‐6* were quantified by RT‐qPCR. (B–C) BV2 cells and primary microglia were pretreated with the indicated inhibitors for 1 h, after which the medium was aspirated. The cells were then incubated with or without JEV‐ApoBDs diluted in inhibitor‐containing medium for 6 h. The relative mRNA levels of *Ccl5*, *Il‐1β* and *Il‐6* were quantified. (D) Schematic diagram of the separation of JEV‐ApoBDs, dead cells, free virions and ApoBD‐free sup. Primary sup: primary supernatant; ApoBD‐free sup: ApoBD‐free supernatant. (E) BV2 cells and primary microglia were separately incubated with or without the aforementioned components for 6 h. The relative mRNA levels of the indicated genes were determined. (F) BV2 cells and primary microglia were separately incubated with JEV‐ApoBDs that were either UV‐irradiated or non‐UV‐irradiated. After 6 h, the cells were harvested to determine the relative mRNA levels of the indicated genes. Data are presented as mean ± SD from at least three biological replicates. Statistical analysis was performed using two‐tailed Student's *t*‐test (A) or one‐way ANOVA followed by Dunnett's post‐hoc test (B, C, E, F). *p < 0.05; **p < 0.01; ***p < 0.001; ****p < 0.0001; ns, not significant. Schematic diagram in (D) was created using BioRender (https://www.biorender.com).

Neuronal apoptosis is known to trigger the inflammatory activation of microglia (Fan et al. 2025; Xiao et al. 2025), and emerging evidence has shown that JEV‐loaded small EVs of non‐neuronal origin can elicit a neuroinflammatory response upon crossing the BBB (Xiong et al. 2025). However, whether ApoBDs released by JEV‐infected neurons exhibit similar inflammatory potential remains to be determined. Our findings revealed that both BV2 cells and primary microglia exposed to ApoBDs markedly upregulated the transcription of inflammatory cytokine genes, such as *Ccl5*, *Il‐1β*, and *Il‐6* (Figure 5A). The magnitude of the pro‐inflammatory responses exhibited a positive correlation with ApoBDs concentration, as mirrored by the progressive decline in mRNA levels of inflammatory cytokines upon serial dilution of ApoBDs (Figure S5A). Furthermore, this transcriptional activation was accompanied by the pronounced release of these inflammatory mediators into the cell culture supernatants (Figure S5B). To extend our findings to a more physiologically relevant setting, we treated primary microglia with ApoBDs derived from JEV‐infected primary mixed neural cultures, and these ApoBDs recapitulated the robust pro‐inflammatory responses observed with N2a‐derived ApoBDs (Figure S5C). Notably, STS‐induced ApoBDs from N2a cells also provoked microglial inflammatory responses (Figure S5D), whereas those generated by ABT‐263 treatment did not (Figure S5E), indicating that the pro‐inflammatory activity of ApoBDs is determined by the specific apoptotic stimulus.

To clarify whether the induction effect of ApoBDs on microglia activation is contributed by their internalization, Cyto D and dynasore were utilized to inhibit the endocytosis process in microglia following their co‐incubation with ApoBDs. Our results indicated that treatment with Cyto D and dynasore significantly decreased the mRNA levels of inflammatory cytokines in ApoBD‐stimulated microglia (Figure 5B,C). Although MβCD slightly suppressed microglial uptake of ApoBDs (Figure 4E), its specific cholesterol depletion unexpectedly triggered microglial hyperactivation even in the absence of ApoBDs (Figure S5F). Hence, MβCD was unsuitable for exploring the interplay between phagocytosis and inflammation in microglia. Collectively, these findings suggest that internalization of ApoBDs produced by JEV‐infected neurons contributes to the elicitation of inflammatory responses in microglia.

To gain deeper insight into the ability of ApoBDs to induce inflammatory responses in microglia, we isolated several components from JEV‐infected N2a cells, including free virions, dead cells, ApoBDs, and ApoBD‐free supernatant (Figure 5D). Prior to treating microglia with these four components, the viral genomic RNA abundance within each was normalized to an equivalent level. The results revealed that ApoBDs possessed the strongest potential to stimulate inflammatory responses, followed by ApoBD‐free supernatant, while free virions exerted minimal such effect (Figure 5E). Furthermore, to determine whether viral infection is necessary for ApoBDs to provoke inflammation, ApoBDs were subjected to UV irradiation for virus inactivation (Figure S5G). As shown in the results, both UV‐irradiated and non‐irradiated ApoBDs induced comparable levels of inflammatory cytokines in microglia (Figure 5F), suggesting that the inflammatory stimulatory property of ApoBDs is independent of viral infection. Together, these data underscore that endogenous damage‐associated molecular patterns (DAMPs) carried by ApoBDs, rather than viral components, act as the key mediators of microglial inflammatory activation.

### 3.6 ApoBDs Generated by JEV‐Infected Neurons Drive Microglial Activation via the TLR2/TLR4–NF‐κB Axis

**FIGURE 6:**
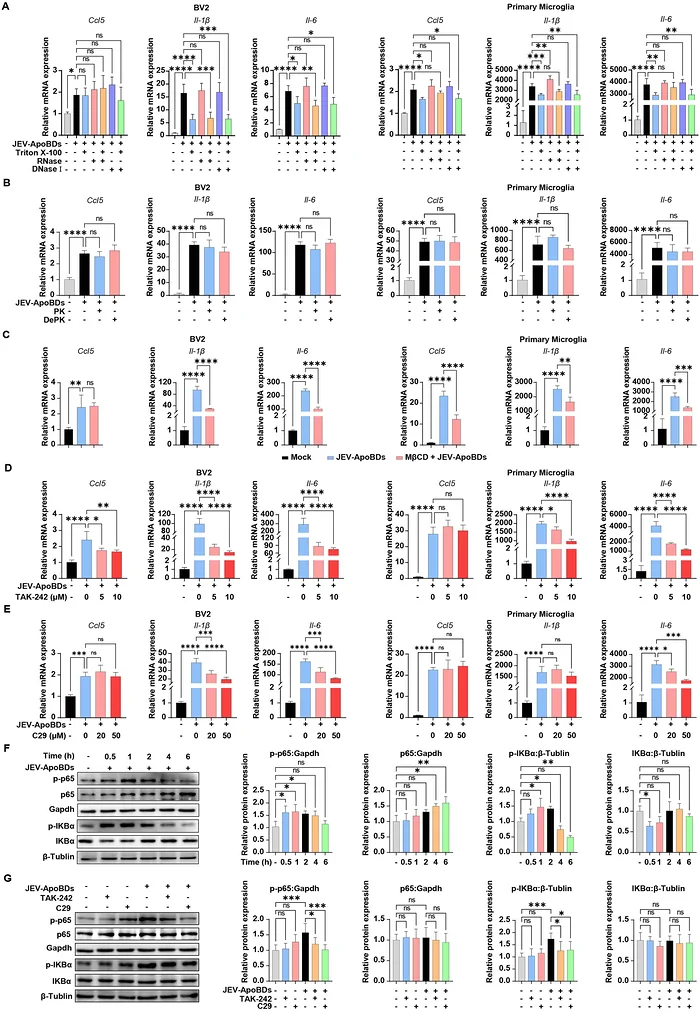
JEV‐ApoBDs derived from JEV‑infected N2a cells activate microglia via the TLR2/TLR4–NF‑κB axis. (A–C) JEV‐ApoBDs pretreated with Triton X‑100, RNase, DNase I, proteinase K (PK), heat‑inactivated proteinase K (DePK), or MβCD were incubated with BV2 cells or primary microglia for 6 h. The relative mRNA levels of the indicated genes were determined. (D–E) Following an 1 h pretreatment with the indicated inhibitors, BV2 cells and primary microglia were incubated with or without JEV‐ApoBDs in inhibitor‐containing medium for 6 h, after which the relative mRNA expression of *Ccl5*, *Il‐1β*, and *Il‐6* was determined. (F) BV2 cells were treated with JEV‑ApoBDs for 0.5, 1, 2, 4 and 6 h. Cell lysates were subjected to Western blotting for p65, phosphorylated p65 (p‑p65), IκBα, and phosphorylated IκBα (p‑IκBα). Bar graphs show the densitometric quantification of the protein bands relative to loading controls. (G) BV2 cells were pretreated with TAK‑242 (10 µM) and C29 (50 µM) for 1 h, then exposed to JEV‑ApoBDs for 2 h. Representative Western blots and densitometric quantification of the indicated proteins are shown. Data are presented as mean ± SD from at least three biological replicates. One‑way ANOVA with Dunnett's (A–F) or Tukey's (G) post hoc test was used for statistical analysis. *p < 0.05; **p < 0.01; ***p < 0.001; ****p < 0.0001; ns, not significant.

To explore the mechanism by which ApoBDs induce inflammation in microglia, we initially identified the specific DAMPs enriched in JEV‐elicited neuronal ApoBDs that engage the microglial activation. ApoBDs directly digested with RNase or DNase I provoked inflammatory responses in microglia comparable to those induced by untreated ApoBDs (Figure 6A). Conversely, pre‐treatment of ApoBDs with Triton X‐100 significantly weakened their pro‐inflammatory capacity. Additionally, subsequent digestion of intraluminal nucleic acids by RNase or DNase I following membrane permeabilization did not further suppress inflammatory gene transcription. Furthermore, ApoBDs exposed to proteinase K retained a robust pro‑inflammatory activity, indicating that their stimulatory capacity was not substantially affected by protein degradation (Figure 6B). Given the membrane‐disrupting property of Triton X‐100, we hypothesized that ApoBD‐associated lipids, instead of proteins or nucleic acids, serve as the pivotal mediators driving microglial activation. To test this, we treated ApoBDs with MβCD and assessed their residual inflammatory stimulatory capacity. As anticipated, microglia stimulated with lipid raft‐disrupted ApoBDs exhibited markedly downregulated expression of inflammatory genes (Figure 6C).

Toll‐like receptors 2 and 4 (TLR2/TLR4) are well‐documented sensors for a variety of lipid species, including oxidized phospholipids and fatty acids (Hwang et al. 2016; Miller et al. 2011). Previous studies have highlighted the critical role of the TLR2/TLR4 and NF‐κB pathway in mediating neuroinflammation during JEV infection (Ashraf et al. 2021; Han et al. 2014; Zhao et al. 2022). Accordingly, we sought to uncover whether TLR2/TLR4 and their downstream NF‐κB signalling cascade function as key effectors of microglial activation in response to ApoBDs derived from JEV‐infected neurons. TAK‐242, a selective TLR4 inhibitor, and C29, a specific TLR2 antagonist, were applied to block receptor signaling during JEV‐ApoBD stimulation. In BV2 cells, TAK‐242 potently inhibited the transcriptional upregulation and secretion of Ccl5, Il‐1β, and Il‐6 (Figure 6D). In primary microglia, although *Ccl5* mRNA levels were not significantly altered, TAK‐242 still markedly reduced the expression of *Il‐1β* and *Il‐6*, as well as the secreted protein levels of all three cytokines (Figures 6D and S6). Similarly, TLR2 antagonism attenuated the transcription and secretion of these inflammatory cytokines (Figures 6E and S6). To verify the convergence of TLR2/TLR4 signalling on NF‑κB activation, we analyzed the phosphorylation status of p65 and IκBα by Western blotting. Time‐course analysis revealed that ApoBDs induced a rapid and transient activation of the NF‐κB (Figure 6F). Specifically, the phosphorylation levels of both p65 and IκBα were significantly elevated between 0.5 and 2 h, followed by a subsequent decline. However, treatment of either TAK‑242 or C29 significantly abrogated the ApoBD‑induced phosphorylation of p65 and IκBα at 2 h post‑stimulation (Figure 6G). At this timepoint, total p65 and IκBα protein levels remained unchanged across all treatment groups. Overall, these results demonstrated that ApoBDs derived from JEV‐infected neurons activate microglia via the TLR2/TLR4–NF‐κB axis.

### 3.7 Neuron‐Produced ApoBDs Contribute to JEV‐Induced Encephalitis

**FIGURE 7:**
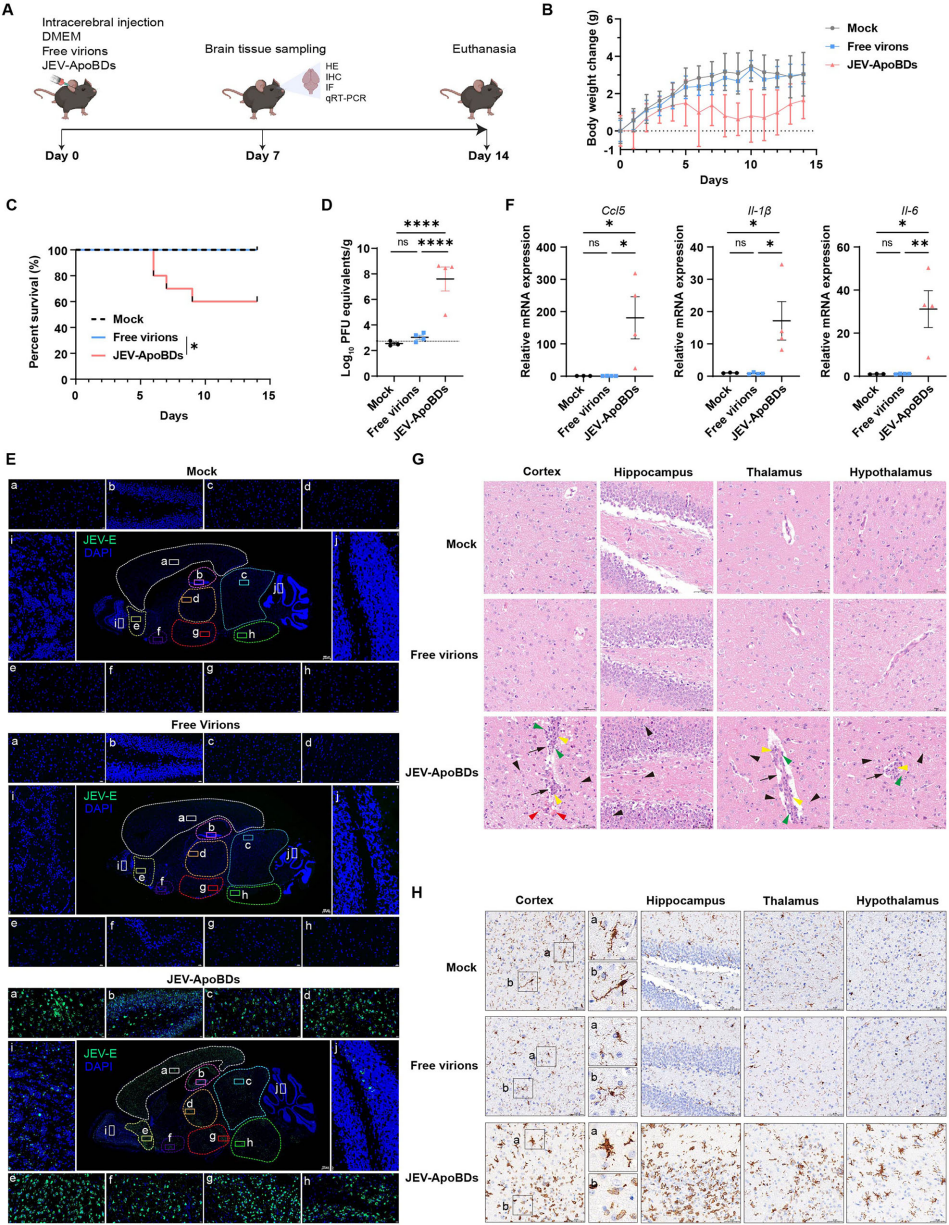
ApoBDs produced by JEV‐infected neurons augment neuroinflammation and neuronal injury. (A) Schematic diagram of mice treatment. (B) Body weight changes of mice across different treatment groups (Mock, *n* = 5; Free virions, *n* = 10; JEV‐ApoBDs, *n* = 10). (C) The survival analysis of mice receiving different treatments (Mock, *n* = 5; Free virions, *n* = 10; JEV‐ApoBDs, *n* = 10). (D) Viral load in mouse brains was determined by Taqman RT‐qPCR, and the calculated value was equivalent to the PFU (Mock, *n* = 3; Free virions, *n* = 4; JEV‐ApoBDs, *n* = 4). (E) Observation of JEV distribution in mouse brains by immunofluorescence assay. Dashed regions: white (a), neocortex; pink (b), hippocampus (solid pink box: dentate gyrus); cyan (c), mesencephalon (solid box: superior colliculus); orange (d), thalamus (solid box: medial nuclear group); yellow (e), anterior olfactory cortex; purple (f), ventral basal forebrain; red (g), hypothalamus (solid box: mammillary nuclear group); green (h), pons (solid box: pontine tegmental reticular nucleus); box (i) is located in the olfactory bulb; box (j) is located in the cerebellum. Scale bar of whole brain: 500 µm. (F) The mRNA levels of *Ccl5*, *Il‐1β* and *Il‐6* in mouse brains were detected by RT‐qPCR, and the results were normalized to *β‐actin* (Mock, *n* = 3; Free virions, *n* = 4; JEV‐ApoBDs, *n* = 4). (G) HE staining‐based assessment of pathological lesions in mouse brain sections. Black arrows: vascular cuffing; red arrowheads: red blood cells (RBCs); green arrowheads: lymphocytes; yellow arrowheads: monocytes; black arrowheads: degenerated neurons. Scale bar: 50 µm. (H) IHC staining of mouse brain sections identifing Iba1‐positive microglia. Scale bar: 50 µm. Log‐rank test was used for survival analysis (C), and one‐way ANOVA followed by Tukey's post hoc test was employed for the analysis of (D, F). *p < 0.05; **p < 0.01; ***p < 0.001; ****p < 0.0001; ns, not significant. Schematic diagram in (A) was created using BioRender (https://www.biorender.com).

To investigate the contribution of ApoBDs to the *in vivo* pathogenesis of JEV, mice were intracerebrally administered free virions or ApoBDs derived from JEV‐infected N2a cells at an equivalent amount of viral genome, with the mock group receiving an equal volume of DMEM (Figure 7A). During the 14‐day observation period, ApoBD‐injected mice exhibited consistently lower body weight gain compared to the other groups. Their body weight decreased from day 6 to day 11 but began increasing from day 12, as some mice recovered from JEV infection. Conversely, the free virions‐injected mice showed body weight changes similar to the mock group (Figure 7B). In accordance with the trend of body weight changes, mice administered ApoBDs began dying on day 6, ultimately reaching a 40% mortality, whereas no mortality was observed in the free virions and mock‐infected group (Figure 7C).

Furthermore, the viral loads in the mouse brain were determined using both RT‐qPCR and immunofluorescence assay. The free virions‐injected group presented an extremely low level of viral load in the cerebral tissue, whereas the ApoBD‐injected group showed a significant increase in cerebral viral load, peaking at an equivalent of 10^8^ PFU/g (Figure 7D). Consistently, immunofluorescence analysis revealed a pronounced signal of the JEV E protein in the brains of mice inoculated with ApoBDs, while almost no positive signal was detected in the brains of mice injected with free virions (Figure 7E). Additionally, the results demonstrated that JEV was extensively disseminated throughout the entire mouse brain. Specifically, the neocortex, hippocampus, and anterior olfactory cortex were identified as the predominant infection sites; additional key infection areas included the anterior nuclear group, medial nuclear group, and reticular nuclear group of the thalamus, the ventral basal forebrain, the mammillary nuclear group of the hypothalamus, the superior colliculus of the mesencephalic tectum, and the reticular nucleus of the pontine tegmentum, among others. Notably, the virus was also abundant in the olfactory bulb, whereas the cerebellum exhibited minimal infection. Further assessment of apoptosis in mouse brain tissues showed that cleaved caspase‐3 was exclusively observed in the ApoBD‐injected group, exhibiting co‐localization with both neurons and microglia (Figure S7A,B).

Since neuroinflammation stands as a defining hallmark of JEV infection within the CNS (Li et al. 2015; Zhu et al. 2023), we evaluated the levels of pro‐inflammatory cytokines in the mouse brains. The results showed that the injection of free virions did not elicit the expression of *Ccl5*, *Il‐1β*, and *Il‐6*, while ApoBD‐injected mice exhibited a significant upregulation of these cytokines in their brain tissue (Figure 7F). Concurrently, ApoBDs‐challenged mice exhibited markedly enhanced expression of the inflammatory markers Nlpr3, Asc, and pro‐Il‐1β in brain regions with high viral loads, such as the cortex, hippocampus, and thalamus, but these signals were absent in either the mock or the free virion group (Figure S7C,D,E). To verify the neuroinflammatory injury, histopathological analysis of brain lesions was conducted. This analysis confirmed that, in mice administered ApoBDs, hippocampal cells displayed disrupted laminar organization, disorganized arrangement, blurred nucleoli in partial cells, and heterogeneous cytoplasmic staining (Figure 7G). Furthermore, other cerebral regions exhibited extravasation of red blood cells, increased eosinophilic reactivity, glial proliferation, neuronal degeneration, and perivascular cuffing characterized by the infiltration of lymphocytes and monocytes. However, no obvious histopathological changes were observed in the brains of free virion‐ and mock‐infected mice. Microglia serve as important drivers of the inflammation process. Our IHC staining results revealed significant microglial proliferation in ApoBD‐injected mouse brains (Figure 7H). These microglia exhibited an amoeboid morphology characterized by enlarged somata, diminished branches, and retracted processes, with a subset even displaying a short rod‐like appearance, indicative of a highly activated phenotype. In comparison, the brains of free virion‐infected mice displayed no notable discrepancies in microglial density and morphological features. Taken together, these findings demonstrate that ApoBDs potentiate neuroinflammation and reinforce the pathogenicity of JEV, highlighting their critical role in exacerbating JEV‐associated neurological disease.

## 4 Discussion

It has been well‐documented that JEV primarily targets neurons and induces their apoptosis (Wongchitrat et al. 2019; Yang et al. 2009). Building on this established knowledge, our study extended this understanding by demonstrating that these JEV‐induced apoptotic neurons release ApoBDs encapsulating infectious JEV particles. This phenomenon mirrors the viral shuttle role of ApoBDs (Atkin‐Smith et al. 2020; Gao et al. 2023b; Bukong et al. 2014; Bello‐Morales and López‐Guerrero 2018), but to our knowledge, this mechanism has not been described in a neurotropic flavivirus. The significantly enhanced infectivity of ApoBDs derived from JEV‐infected neurons, compared with free virions, carries profound biological implications for JEV pathogenesis—a feature intrinsic to the ApoBD‐encapsulated JEV rather than stemming from contaminating free virions. Furthermore, the faster infection kinetics of ApoBDs likely reflect more efficient cellular entry pathways. This aligns with previous studies, which demonstrated that pathogen‐containing EVs generally exploit phagocytic pathways for rapid and efficient internalization by recipient cells (Chahar et al. 2015; Meier et al. 2002; Nanbo et al. 2013), highlighting a conserved evolutionary advantage of vesicle‐mediated viral entry in evading host immune defenses.

Our detailed investigation into the microglial uptake mechanisms of ApoBDs revealed a predominant role for actin‐dependent phagocytosis and dynamin 2‐dependent endocytosis, with minimal contribution from the CIE pathway. This finding is in contrast with the well‐characterized routes by which free JEV virions enter non‐neuronal cells via CME and N2a cells via CIE, respectively (Liu et al. 2017; Nawa et al. 2003; Yang et al. 2013), underscoring the pathway specificity of vesicle‐mediated viral delivery. Intriguingly, a high degree of dependence on dynamin 2 was observed solely in primary microglia, and not in BV2 cells, suggesting cell‐type‐specific differences in endocytic machinery. This phenomenon can be attributed to functional heterogeneity between primary cells and immortalized cell lines, a critical caveat when extrapolating *in vitro* results to *in vivo* scenarios.

The present study clearly identifies JEV‐loaded ApoBDs as the most potent effector contributing to this inflammatory activation, followed by ApoBD‐free supernatant containing small vesicles, and free virions as the weakest factor, indicating that EVs play a more critical role in neuroinflammation, especially ApoBDs. This finding also supports the notion that the vesicle populations released by nerve cells upon JEV infection are likely the key factors activating microglia, rather than direct viral infection. Consistently, UV‐irradiated ApoBDs retained their ability to induce microglial inflammation, indicating that the infectious virus is not required for this pro‐inflammatory effect. A distinction should be made between JEV‑loaded and JEV‑free ApoBDs regarding their contributions to JE pathogenesis. ApoBDs harbouring JEV play a dual role, as they deliver infectious virus to recipient cells, sustaining viral spread and fuelling the persistent production of new ApoBDs, while also carrying DAMPs that activate microglia. In contrast, JEV‑free ApoBDs contribute mainly to neuroinflammation without mediating viral dissemination. Both ApoBD populations potently drive neuroinflammation, as we found that the lipid constituents of ApoBDs serve as critical mediators of microglial activation. Since JE lethality is ultimately a consequence of neuroinflammatory damage (Chen et al. 2012; Sharma et al. 2021), the synergistic action of the two populations is a contributing factor to disease severity, with the JEV‐loaded ApoBDs acting as the initial fuse that ignites the entire pathogenic process.

An intriguing finding in our study is the contrasting ability of ApoBDs generated by different apoptotic triggers to activate microglia. Specifically, while both JEV infection and STS treatment induced ApoBDs capable of robustly stimulating pro‐inflammatory responses in microglia, ApoBDs derived from ABT‐263‐treated neurons failed to elicit such activation. Indeed, a subset of senescent or dysfunctional neurons in adult brain tissue undergoes apoptosis, while during development, neurons experience synaptic pruning in the process of neural circuit establishment—neither of which activates microglia (Boada‐Romero et al. 2020; Brown and Neher 2014; Doran et al. 2020; Zhao et al. 2021). This discrepancy likely stems from the distinct mechanisms of cell death induction. ABT‐263 triggers apoptosis via direct Bcl‐2 inhibition, mimicking a physiological, ordered clearance process. Such vesicles likely sequester cellular contents without exposing potent immunostimulatory ligands, or they may retain surface markers that enforce immune tolerance. Conversely, JEV infection and STS treatment represent severe pathological insults that induce rapid, stress‐associated cell death. These conditions promote the leakage or enrichment of DAMPs and specific pro‐inflammatory lipids into the forming ApoBDs. Consequently, the microglial response is not merely a reaction to phagocytosis per se, but a specific recognition of the danger cargo borne by vesicles generated under pathological stress. This distinction highlights that not all ApoBDs are functionally equivalent, and the nature of the apoptotic stimulus plays a pivotal role in determining the quality or molecular cargo of the resulting ApoBDs.

Using a mouse model of JE, the augmented neuronal damage and increased lethality associated with ApoBDs containing JEV highlight their direct clinical relevance. Additionally, mice administered JEV‐loaded ApoBDs exhibit higher mortality than those receiving free virions, a phenomenon explained by robust CNS viral replication coupled with excessive neuroinflammation; both factors are widely recognised as major mediators of JE fatalities (Li et al. 2015). ApoBDs not only retain a subset of membrane proteins from their parental cells but also can sequester JEV particles within their lumen alongside other biomacromolecules, including nucleic acids and apoptotic proteins (Kumar et al. 2020), which enables ApoBDs to mediate JEV transmission via a non‐canonical pathway that differs fundamentally from the receptor‐dependent single‐cell infection mode of free virions. Specifically, ApoBDs can be internalized by recipient cells through phagocytic uptake, thereby delivering encapsulated JEV directly into the cytoplasm—bypassing the rate‐limiting step of free virion binding to specific cell surface receptors. This transmission mode confers three critical advantages: first, it evades humoral immune surveillance. The membrane structure of ApoBDs can shield JEV surface antigens, preventing recognition and neutralization by antibodies *in vivo*, thereby significantly prolonging the survival time of the virus (Raab‐Traub and Dittmer 2017). Second, it improves infection efficiency. A single ApoBD can encapsulate multiple JEV particles, which are simultaneously released upon internalization by target cells, rapidly initiating progeny virus replication and forming infection foci (Urbanelli et al. 2019). Third, it facilitates the establishment of viral infection in non‐susceptible cells (Urbanelli et al. 2019). Beyond facilitating viral transmission in the CNS, the ApoBD‐mediated inflammatory amplification effect is attributable not only to the DAMPs‐PRRs axis, but also to its recruitment of more immune cells to infiltrate brain tissue, intensified immune cell attack on infected neurons, and concomitant increases in vascular permeability and BBB disruption. From a different perspective, it is worth considering whether this effect is triggered by the direct microglial engulfment of intact JEV‑ApoBDs or results from the passive release of pro‑inflammatory cargo following the gradual lysis of these vesicles in the extracellular space. Mechanistically, these two possibilities are not mutually exclusive and may act synergistically. First, *in vitro* data clearly demonstrate that the inhibition of ApoBD endocytosis significantly attenuates the inflammatory responses in microglia, supporting the notion that direct phagocytosis triggers inflammation. Concurrently, the pro‐inflammatory activity of ApoBDs is dependent on their lipid constituents. This implies that even if ApoBDs are partially lysed *in vivo*, the released or exposed lipid‐based DAMPs could still directly activate surrounding microglia, thereby establishing a pro‐inflammatory microenvironment. Under the complex physiological conditions *in vivo*, a fraction of ApoBDs may be engulfed by microglia to activate inflammatory signalling, while another fraction may gradually degrade within the extracellular matrix, releasing their contents and exacerbating local inflammation in a paracrine manner. Moreover, JEV released upon ApoBDs disintegration would rapidly initiate broader infection in surrounding cells, further amplifying viral dissemination and tissue damage. This dual mode of action not only exacerbates neuroinflammation but also propagates JEV pathogenicity. Future studies employing *in vivo* tracing techniques to distinguish between intact and degraded ApoBDs will be valuable to further elucidate the relative contributions of these two pathways at different stages of the disease course. In contrast to ApoBDs harbouring JEV, free virions exhibited substantially lower infectivity and pathogenicity. One key reason is that free virions are vulnerable to humoral immune clearance. Unlike ApoBD‐encapsulated JEV, the surface antigens of free virions are fully exposed to the extracellular milieu, making them prime targets for neutralizing antibodies (Xiong et al. 2025). This rapid antibody‐mediated neutralization drastically curtails the window of opportunity for free virions to bind and infect target cells. Another major factor is that free virions exhibit inherently low single‐cell infection efficiency. Successful infection by a single free virion requires sequential, rate‐limiting steps: attachment, penetration, uncoating, replication. Each of these steps is susceptible to host antiviral defenses, resulting in a low probability of productive infection and limited progeny virus production (Greseth and Traktman 2022; Jones et al. 2021; Thiyagarajah et al. 2023). In light of recent findings that JEV‐loaded sEVs from peripheral cells facilitate JEV crossing the BBB and subsequent infection (Xiong et al. 2025), combined with the role of neuron‐derived ApoBDs in JEV pathogenesis revealed in this study, it is thus reasonable to hypothesize that EV‐mediated viral hijacking may represent a primary mechanism of JEV dissemination *in vivo*.

Based on the above results, limitations emerge that warrant attention in future research. Here, we have validated the presence of several ApoBD markers by Western blotting, and further confirmed the localization of cleaved caspase‑3 on ApoBDs by IEM. Hmgb1 and Lamin‑B1 were barely detectable in ApoBDs. This aligns with the current understanding that apoptosis induces histone hypoacetylation, which tightly retains Hmgb1 on chromatin (Scaffidi et al. 2002), while Lamin‑B1 is proteolytically cleaved by caspases during apoptosis, in contrast to necrosis where it remains intact (Bortul et al. 2001; Lüthi and Martin 2007; Slee et al. 2001). Their near‑absence thus confirms the apoptotic origin of our ApoBD preparations and excludes significant contamination by necrotic debris or intact nuclei. CD47 is a well‐known transmembrane protein localized on the plasma membrane. The substantial enrichment of CD47 in the ApoBD fractions compared to intact N2a cells indicates that our isolation process successfully captured membrane‐derived vesicles, consistent with the biogenesis of ApoBDs through plasma membrane blebbing during apoptosis. In addition, we characterized Annexin V positivity and size distribution. Nevertheless, a more comprehensive molecular characterization is warranted. Future studies should employ proteomics and lipidomics to fully define the molecular signatures of neuron‑derived ApoBDs under JEV infection and to differentiate them from ApoBDs generated by other apoptotic stimuli. Such analyses would not only distinguish ApoBDs from other extracellular vesicle subtypes, but also pinpoint the specific effectors that govern their pro‑inflammatory potency and viral infection efficiency, thereby providing a more robust foundation for understanding their pathophysiological roles and for developing targeted interventions. Most mechanistic insights have been obtained from *in vitro* pharmacological experiments, and genetic manipulation approaches merit further application to validate these findings. Meanwhile, confirming these observations in more physiologically relevant models (e.g., brain organoids and conditional knockout mice) will be essential to corroborate the *in vivo* relevance of pathogenesis mediated by ApoBDs. Our study focused primarily on microglia and neurons; the role of other CNS‐resident cells (e.g., astrocytes and oligodendrocytes) in ApoBDs uptake, signalling, and subsequent contribution to neuroinflammation remains unexplored. In addition, *in vivo* experiments in mice demonstrate the pathogenic role of neuron‐derived ApoBDs, but the clinical relevance of these findings to human JE remains to be established. Cerebrospinal fluid (CSF) is the most direct and informative specimen for detecting CNS‑derived vesicles, owing to its contact with brain parenchyma and its reflection of ongoing neuroinflammation and neurodegeneration. However, acquiring such samples poses considerable challenges, primarily due to the limited number of confirmed JE cases with accessible biospecimens, alongside ethical and practical constraints. Furthermore, the systematic purification of ApoBDs directly from brain tissue still encounters technical hurdles. While differential centrifugation and FACS‐based sorting have been applied to isolate ApoBDs from cultured cells or biological fluids, their application to solid neural tissue introduces substantial complexities, including inefficient tissue dissociation, co‐sedimentation with myelin and cellular debris, and low yields of intact vesicles (Pérez‐González et al. 2017). Ultracentrifugation, the current gold standard for EV isolation, also suffers from co‐isolation of contaminants and potential physical damage to large vesicles such as ApoBDs (Vechetti 2019). Therefore, developing standardized, tissue‐compatible isolation protocols for ApoBDs is urgently required. Despite these limitations, our findings significantly advance understanding of JEV pathogenesis and provide a framework for developing novel therapeutic strategies. Specifically, targeting ApoBD formation via selective block of apoptotic blebbing without disrupting antiviral immunity (Atkin‐Smith et al. 2020), or inhibiting microglial ApoBD uptake using inhibitors like dynasore, could potentially reduce viral spread and mitigate neuroinflammation. Additionally, ApoBDs hold promise as a novel biomarker for the progression and severity of JE, given that their circulating levels have been linked to disease outcomes in other disease models (Díaz‐Maroto et al. 2025; Serrano‐Heras et al. 2020).

**FIGURE 8:**
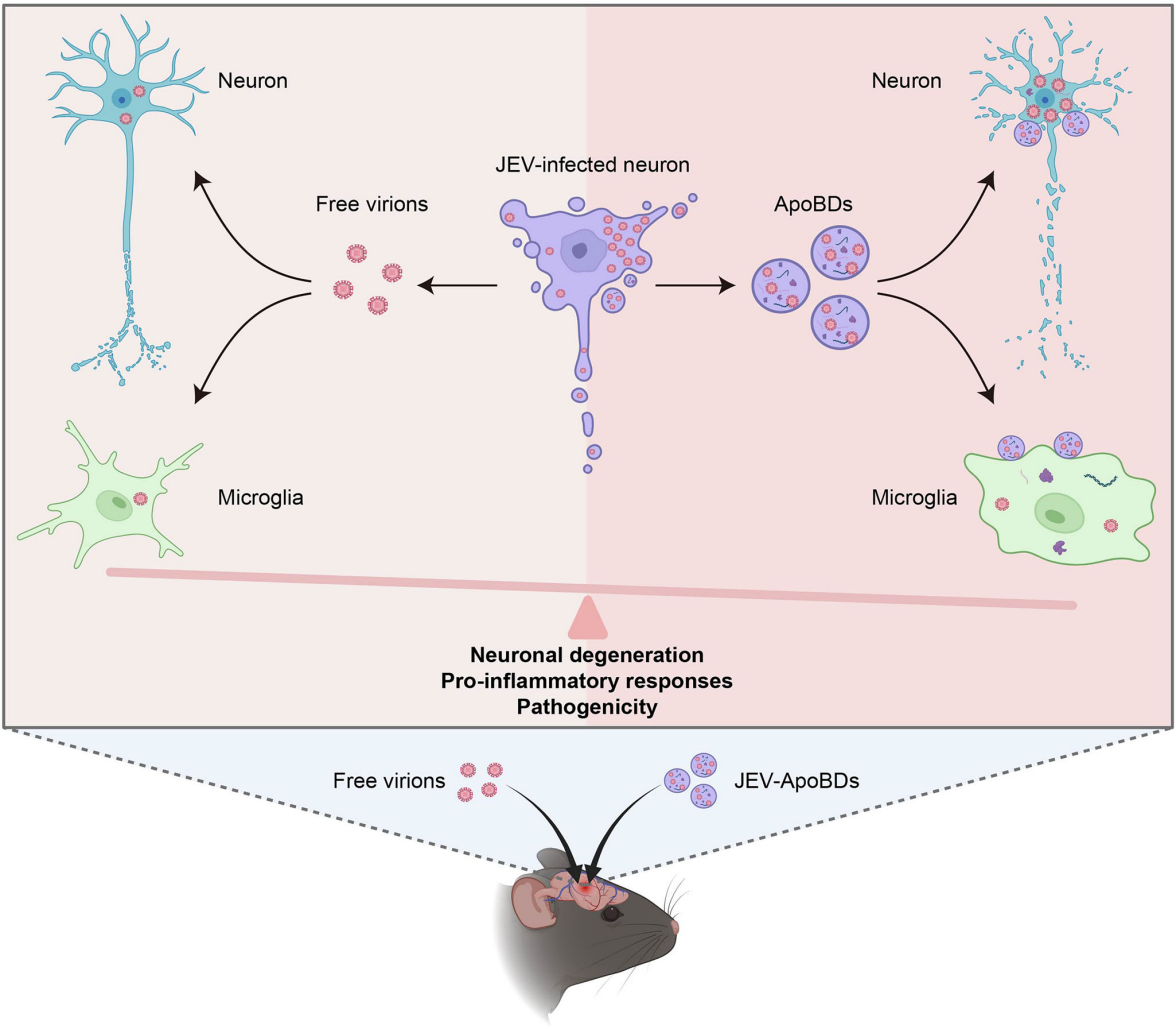
Schematic diagram of neuron‐derived ApoBDs facilitating JEV infection and pathogenicity. Neuronal‐derived ApoBDs potentiate JEV infection establishment in the CNS. Free virions and ApoBD‐encapsulated virions were liberated into the extracellular space from infected neurons. Compared to free virions, both neurons and microglia are more susceptible to ApoBD‐encapsulated JEV infection, thus exacerbating neuronal degeneration. In addition, ApoBDs synergize with JEV infection to elicit potent inflammatory activation in microglia. Schematic diagram was created using BioRender (https://www.biorender.com).

In conclusion, our study identifies ApoBDs as critical mediators of viral dissemination and neuroinflammation in during JEV infection in the CNS (Figure 8), expanding the growing repertoire of EV‐dependent viral pathogenesis mechanisms. These findings not only bridge important gaps between JEV pathogenesis and EV functions but also provide a novel conceptual framework for understanding how neurotropic viruses exploit apoptotic processes to enhance their infectivity and pathogenicity. By uncovering the dual role of ApoBDs in promoting viral transmission and triggering neuroinflammation, our work offers a promising foundation for developing targeted therapeutic interventions against JE—an urgent need given the lack of specific antiviral treatments. As EV‐based therapies and diagnostic technologies continue to advance (Ipinmoroti and Matthews 2020; Kumar et al. 2020; Rodrigues et al. 2018), the role of ApoBDs in infectious diseases, particularly neurotropic viral infections like JE, will grow increasingly important for translating basic research discoveries into clinical applications that improve patient outcomes.

## Author Contributions

**Shengbo Cao**: conceptualization, funding acquisition, resources, supervision, visualization, writing – original draft. **Xiaowei Nan**: investigation, software, visualization. **Youhui Si**: resources, supervision. **Ting Wang**: data curation, formal analysis. **Junyao Xiong**: investigation, methodology. **Chengjie Yang**: project administration, resources. **Jing Ye**: conceptualization, funding acquisition, resources, supervision, validation, visualization, writing – original draft, writing – review and editing. **Chuan Zeng**: conceptualization, methodology, software, data curation, investigation, visualization, formal analysis, project administration, writing – original draft, writing – review and editing, resources. **Bibo Zhu**: supervision, resources. **Shuo Zhu**: resources, project administration.

## Ethics Statement

The animal study was reviewed and approved by The Scientific Ethic Committee of Huazhong Agriculture University (HZAUMO‐2025‐0006). Written informed consent was obtained from the owners for the participation of their animals in this study.

## Conflicts of Interest

The authors declare no conflicts of interest.

## Supporting information




**Supporting Information**: jev270366‐sup‐0001‐FigureS1‐S7.docx

## Data Availability

The data that supports the findings of this study are available in the supplementary material of this article.
