# Supplementary material for: Neuronal Apoptotic Bodies Facilitate Japanese Encephalitis Virus Infection and Pathogenicity
Source: J Extracell Vesicles. 2026 Sep 1;15(9):e70366. doi: 10.1002/jev2.70366 (PMC13533795; doi:10.1002/jev2.70366)
Supplement: Supplementary file 1 [file JEV2-15-e70366-s001.docx]

**Figure S1** JEV replication kinetics and apoptosis induction in N2a Cells. (A-C) N2a cells were infected with JEV at an MOI of 1 or 5, respectively. At 12, 24, 36, and 48 hpi, the cells were fixed with 4% paraformaldehyde, followed by immunofluorescence staining with anti-JEV E mAb. Scale bar: 100 μm (A). The supernatant was collected to quantify viral titers using plaque assay (B). Cell viability was assessed by CCK-8 assay (C). (D-E) N2a cells were infected with JEV (1 MOI). Cleaved caspase-3 (D) and annexin V-mCherry (E) were detected using fluorescence microscopy at 36 and 48 hpi. Scale bar: 100μm. One-way ANOVA with Dunnett’s post-hoc test was performed to compare differences (C). *p < 0.05; **p < 0.01; ***p < 0.001; ****p < 0.0001; ns, not significant.

**Figure S2** Neuronal ApoBDs promote JEV infection. (A) Free virions were isolated by sucrose density gradient centrifugation. The gradient consisted of 20%, 35%, 55%, and 70% sucrose layers. Free virions were located at the interface between the 55% and 70% layers. (B-C) BHK‑21 cells were infected with free virions or JEV‑ApoBDs at equal genomic RNA levels and immunostained for JEV E (green) and DAPI (blue) at the indicated time points (B). The fluorescence integrated intensity of JEV E protein from (B) was quantified (C). Statistical significance was assessed using unpaired two‑tailed Student's t‑test. *p < 0.05, **p < 0.01, ***p < 0.001.

**Figure S3** CCK-8 assay. BV2 cells and primary microglia were incubated with the indicated inhibitors at varying concentrations, after which cell viability was examined via the CCK-8 assay. PM: primary microglia

**Figure S4** Effects of inhibitors on microglial uptake of JEV-ApoBDs. (A-B) BV2 cells and primary microglia were incubated with Dil-labeled JEV-ApoBDs (red) for 3 h in the presence or absence of EIPA and pitstop2 at different concentrations. Cells stained with anti-CD45 antibody (green) and DAPI (blue) were observed under a confocal microscope (A). Scale bar: 100 μm. The uptake efficiency was analyzed (B). (C) Dil-labeled JEV-ApoBDs were pre‑incubated with 20 μg Annexin V for 2 h at RT, and then added to microglia. The uptake efficiency was quantified by flow cytometry. One-way ANOVA with Dunnett’s comparison was used in (B). Unpaired two‑tailed Student's t‑test was used in (C), *p < 0.05, ***p < 0.001.

**Figure S5** JEV-ApoBDs provoke pro-inflammatory responses in microglia. (A) BV2 cells or primary microglia were treated with serially diluted (10‑fold) JEV‑ApoBDs for 6 h, and the mRNA levels of inflammatory genes were assessed by RT‑qPCR. (B) BV2 cells and primary microglia were incubated with JEV‑ApoBDs for 24 h, and culture supernatants were collected. The secreted levels of Ccl5, Il‑1β, and Il‑6 were measured by ELISA. (C) Primary mixed neural cells were infected with JEV at an MOI of 1 for 48 h, and ApoBDs were isolated from the culture supernatants. These ApoBDs were then co‑incubated with primary microglia for 6 h, after which transcript levels of the indicated genes were quantified by RT‑qPCR. (D-E) BV2 cells and primary microglia were treated for 6 h with JEV‑ApoBDs, STS‑ApoBDs (D), or ABT‑263‑ApoBDs (E), and the mRNA levels of *Ccl5*, *Il‑1β*, and *Il‑6* were quantified by RT‑qPCR. (F) BV2 cells and primary microglia were pre-treated with MβCD. Following medium aspiration at 1 h post-treatment, the cells were incubated with JEV-ApoBDs (diluted in inhibitor-supplemented medium) for 6 h, after which the relative mRNA levels of *Ccl5*, *Il-1β*, and *Il-6* were quantified. (G) JEV-ApoBDs were irradiated with UV light (220V, 30W) for 5 min on ice, followed by plaque assay. Data are presented as mean ± SD of at least three biological replicates. Statistical significance was determined by unpaired two‑tailed Student's t‑test (B, C) or one‑way ANOVA followed by Dunnett's (A, F) or Tukey's (D, E) post hoc test. *p < 0.05; **p < 0.01; ***p < 0.001; ****p < 0.0001; ns, not significant.

**Figure S6** TLR2/TLR4 inhibition attenuates JEV-ApoBD‑induced cytokine secretion. BV2 cells and primary microglia were incubated with ApoBDs for 24 h in the presence or absence of 10 μM TAK‑242 or 50 μM C29. Culture supernatants were collected, and the secreted levels of Ccl5, Il‑1β, and Il‑6 were measured by ELISA. Data are mean ± SD from three biological experiments. Statistical significance was determined by one‑way ANOVA with Dunnett's post hoc test. *p < 0.05, **p < 0.01, ***p < 0.001.

**Figure S7** JEV-ApoBDs induce apoptosis and inflammatory activation in the mouse brain. (A-B) Representative immunofluorescence images of cleaved‑caspase‑3 (green) co‑stained with Map2, a neuronal marker (A), or Iba1, a microglial marker (B), in the cortex, hippocampus, thalamus, and hypothalamus of mice injected with DMEM (mock), free virions, or JEV‑ApoBDs. Scale bar: 50 μm or 100 μm (Enlarged views). White and yellow arrowheads indicate cleaved caspase-3⁺/Map2⁺ and cleaved caspase-3⁺/Iba1⁺ cells, respectively. (C-E) Immunofluorescence staining shows Asc (C), Nlrp3 (D), and pro-Il-1β (E) in the indicated brain regions from mice treated as described in (A). Scale bar: 50 μm.
